# CoRe: a robustly benchmarked R package for identifying core-fitness genes in genome-wide pooled CRISPR-Cas9 screens

**DOI:** 10.1186/s12864-021-08129-5

**Published:** 2021-11-17

**Authors:** Alessandro Vinceti, Emre Karakoc, Clare Pacini, Umberto Perron, Riccardo Roberto De Lucia, Mathew J. Garnett, Francesco Iorio

**Affiliations:** 1 Human Technopole, Milan, Italy; 2 Wellcome Sanger Institute, Wellcome Genome Campus, Hinxton, Cambridge, UK

**Keywords:** CRISPR-Cas9 screens, core-fitness genes, cancer dependency, algorithms, benchmark

## Abstract

**Background:**

CRISPR-Cas9 genome-wide screens are being increasingly performed, allowing systematic explorations of cancer dependencies at unprecedented accuracy and scale. One of the major computational challenges when analysing data derived from such screens is to identify genes that are essential for cell survival invariantly across tissues, conditions, and genomic-contexts (core-fitness genes), and to distinguish them from context-specific essential genes. This is of paramount importance to assess the safety profile of candidate therapeutic targets and for elucidating mechanisms involved in tissue-specific genetic diseases.

**Results:**

We have developed CoRe: an R package implementing existing and novel methods for the identification of core-fitness genes (at two different level of stringency) from joint analyses of multiple CRISPR-Cas9 screens. We demonstrate, through a fully reproducible benchmarking pipeline, that CoRe outperforms state-of-the-art tools, yielding more reliable and biologically relevant sets of core-fitness genes.

**Conclusions:**

CoRe offers a flexible pipeline, compatible with many pre-processing methods for the analysis of CRISPR data, which can be tailored onto different use-cases. The CoRe package can be used for the identification of high-confidence novel core-fitness genes, as well as a means to filter out potentially cytotoxic hits while analysing cancer dependency datasets for identifying and prioritising novel selective therapeutic targets.

**Supplementary Information:**

The online version contains supplementary material available at 10.1186/s12864-021-08129-5.

## Background

The ability to perturb individual genes at scale in human cells holds the key to elucidating their function and it is a gateway to the identification of new therapeutic targets across human diseases, including cancer. In this context the CRISPR-Cas9 genome editing system is the state-of-the-art tool [1–3].

Several genome-scale CRISPR-Cas9 single guide RNA (sgRNA) libraries have been designed and are available to date for genetic perturbation screens in human cells, showing significantly improved precision and scale with respect to previous technologies [4–8]. Some of these libraries have been employed in large-scale in-vitro screens assessing each gene’s potential in reducing cellular viability/fitness upon inactivation, across hundreds of immortalised human cancer cell lines [7, 9–12]. This has led to comprehensive identifications of cellular fitness genes, providing a detailed view of genetic dependencies and vulnerabilities existing in cancer cells.

Several sources of bias must be considered when analysing dependency profiles derived from CRISPR-Cas9 screens. These include different guide efficiency and off-target effects [13, 14], genomic features like copy number amplifications [7, 15–17], variable phenotypic penetrance [18], and different experimental settings such as, for example, screening time length and cells’ growth medium [19, 20]. Taken together, these factors contribute to making the analysis of CRISPR-Cas9 screens not trivial, and several tools have been proposed for this task [12, 21–25].

When analysing data from CRISPR-Cas9 screens in functional and translational studies another major computational problem is to classify and distinguish genetic dependencies involved in normal essential biological processes from disease- and genomic-context-specific vulnerabilities.

Identifying context-specific essential genes, and distinguishing them from constitutively essential genes shared across all tissues and cells, i.e. core-fitness genes (CFGs), is also crucial for elucidating the mechanisms involved in tissue-specific diseases. Moving forward,focusing on very well-defined genomic contexts in tumours allows identifying cancer synthetic lethalities that could be exploited therapeutically [26].

Gene dependency profiles, generated via pooled CRISPR-Cas9 screening across large panels of human cancer cell lines, are becoming increasingly available [27, 28]. However, identifying and discriminating CFGs and context-specific essential genes from this type of functional genetics screens remains not trivial.

The Daisy Model (DM) has been recently described for identifying CFGs by jointly analysing data from genetic screens of multiple cancer cell lines. In this approach, sets of fitness genes for each screened cancer cell line are conceptually represented by the petals of a daisy [10]. These have different extents of overlap, but they generally tend to share a common set of CFGs (the core of the daisy). Based on this idea, genes that are essential in most of the screened cell lines are predicted to be CFGs. This approach has been shown to be able to identify CFGs that are enriched for fundamental cellular processes such as transcription, translation, and replication [10]. Nevertheless, in [10] the minimal number of cell lines (3 out of 5 screened) in which a gene should be significantly essential in order to be predicted as CFG, is arbitrarily defined with no indications on how to determine this threshold on a numerically grounded basis when applying the DM to larger collections of screens.

In [11] we have introduced the Adaptive Daisy Model (ADaM): a generalisation of the DM that is able to determine the minimal number of cell lines that should be vulnerable to knocking-out the putative CFGs, i.e. dependent on them, in a semi-supervised manner.

We have also recently proposed an alternative unsupervised approach within the Broad and Sanger Institutes’ Cancer Dependency Map collaboration [29], where data from screening hundreds of cell lines are analysed in a pooled fashion, independently of their tissue of origin. This method builds on the intuition that if a gene is universally essential then it should rank among the top essential genes in most screened models, including those that are the least dependent on it, or generally showing a moderate to weak loss-of-fitness phenotype upon CRISPR-Cas9 targeting.

Finally, a logistic regression based method for classifying genes into CFGs or context-specific essentials has been recently introduced by Sharma and colleagues [30] as part of the CEN-tools suite, using reference sets of essential and non-essential genes for the training phase [31].

Although the number of CRISPR-Cas9 and genome-scale RNAi experiments is increasing rapidly, no robustly benchmarked method to identify sets of CFGs has been devised yet in a unique and easy-to-use software package.

We present CoRe: an R package implementing recently proposed as well as novel versions of algorithms for the identification of CFGs from a joint analysis of multiple genome-wide pooled CRISPR-Cas9 knock-out screens. Furthermore, we present results from a comparison of CoRe’s output (when applied to the largest integrative cancer dependency dataset generated to date [19]) against widely used [10, 31], or more recent [30] sets of CFGs obtained via an alternative approach (which we have also tested on the same recent cancer dependency dataset). We report an increased coverage of prior known human essential genes, new potential core-fitness genes, and lower false positive rates for CoRe’s methods with respect to other state-of-the-art core-fitness sets and available methods. Finally we show that CoRe is computationally more efficient than other methods, and that the CFGs obtained with CoRe could be used in the future as a template classifier of a single screen’s specific essential genes, via supervised classification methods, such as the widely used BAGEL [24].

## Implementation

### Overview of the CoRe package

CoRe implements two methods at two different levels of stringency yielding, respectively, (i) core-fitness essential genes (CFGs) and (ii) common-essential genes (CEGs). Both sets include genes that are essential for cell survival invariantly across tissues and genomic backgrounds and are involved in housekeeping cellular processes, thus are conceptually the same. However, CFGs are identified in CoRe more stringently and in a supervised manner, whereas CEGs are outputted by a less stringent and unsupervised method. These two-level of stringency make CoRe suitable for a variety of use-case scenarios. These range from the robust identification of new human core essential genes (where minimising false positive is essential, thus CFGs should be preferred to CEGs), to filtering out potential cytotoxic candidates when focusing on context-specific essential genes while identifying and prioritising new therapeutic targets (where is more important to minimise the false negatives, thus CEGs should be preferred to CFGs).

**Fig. 1:**
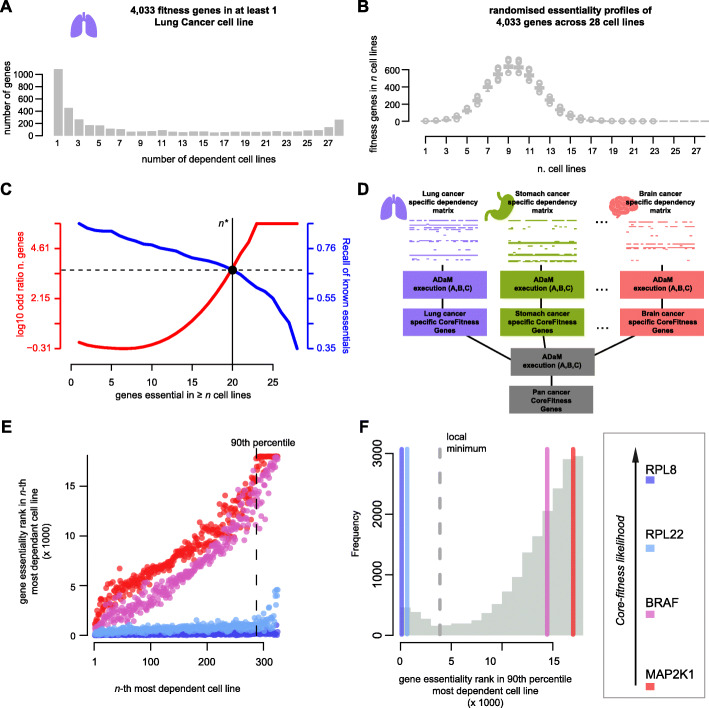
Overview of the methods implemented in CoRe. **A.** Number of fitness genes in fixed numbers of cell lines (CLs) from a lung specific binary cancer dependency matrix (BM). **B.** As for A but considering 1000 randomisations of the lung BM. **C.** ADaM execution on the lung BM: The aim is to identify the minimal number n* of CLs in which a gene should be essential to be considered a lung specific core-fitness essential gene (CFG). All possible *n* values (on the x-axis) are tested. For each *n* the genes essential in ≥ *n* CLs are determined. The Recall of a reference set of CFGs (blue curve, and right y-axis) is computed for this set of genes. At the same time the deviance of expectation of the size of this set of genes is also computed (log_10_ ratio with respect to average value in 1000 permutations of the lung BM - red curve, and left y-axis). The n* value (solid vertical line) is that providing the best trade-off (dashed horizontal line) between the blue and the red curves. **D.** Schematic of the two-step model of ADaM identifying pan-cancer CFGs. The first determines sets of tissue/cancer-type specific CFGs. The second step computes pan-cancer CFGs as those predicted as tissues/cancer-type specific core-fitness genes for at least t* tissues/cancer-types. This is determined as for the n* in C. **E.** Basic assumption of the FiPer method: common-essential genes (CEGs) are always among the top essential genes. 4 example genes are shown. Each point indicates a CL. The coordinate on the x-axis indicates the rank position of the CL when sorting all CLs based on their dependency on the gene under consideration, in decreasing order. The coordinate on the y-axis indicates the rank position of the gene under consideration from sorting all screened genes based on their fitness scores observed in the CL under consideration, decreasingly. Common-essential genes (RPL8 and RPL22) ranks always among the top fitness scores, resulting in an almost flat trend. The vertical dashed line indicates the 90th percentile of dependency on the gene under consideration. **F.** Distribution of all genes’ fitness-rank-positions for the CL at their 90th-percentile of least dependent cell lines, i.e. the dashed vertical line in E). The density of these scores is estimated using a Gaussian kernel and the central point of minimum density is identified. Genes whose score falls below this minimum (i.e. to the left of the gray dashed line) are classified as common-essential by FiPer Fixed

The first and more stringent method implemented in CoRe is the Adaptive Daisy Model (ADaM) [11]: an adaptive version of the Daisy Model (DM) [10] that operates in a cascade of two steps, and it is usable on data coming from large-scale CRISPR-cas9 knock-out screens performed in heterogeneous in-vitro models, for example immortalized human cancer cell lines from multiple tissue lineages (Fig. 1A-D).

The second and less stringent CoRe method, implemented in four different novel variants, is the Fitness Percentile (FiPer) method, which identifies CEGs via a pooled (pan-cancer) analysis of data from large-scale CRISPR-Cas9 knock-out screens, performed in cell lines from multiple tissues/cancer-types﻿  [20] (Fig. 1EF). For each screened cell line, this approach considers the gene rank positions resulting from sorting all screened genes based on their effect on cell viability upon CRISPR-Cas9, i.e. their essentiality, in decreasing order. FiPer then exploits the intuition that CEGs will always rank among the top essential genes for most cell lines, including those for which the fitness reduction is overall less pronounced.

While ADaM takes as input strictly defined binary scores of gene essentiality and it outputs discrete sets of tissue-specific and pan-cancer CFGs, FiPer takes as input quantitative descriptors of gene essentiality and it outputs a unique set of CEGs, also providing a visual means for quickly assessing the tendency of individual genes to be a CEG.

#### The Adaptive Daisy Model

The Adaptive Daisy Model (ADaM) [11] is implemented in the function CoRe.ADaM of CoRe, which takes as input (i) a binary dependency matrix, where rows correspond to genes and columns to samples (screens or cell-lines), with a 1 in position *[i, j]* indicating that the inactivation of the *i*-th gene through CRISPR-Cas9 targeting exerts a significant loss of fitness in the *j*-th sample, i.e. that the *j*-th cell line is dependent on the *i*-th gene; (ii) a reference set of prior known CFGs. Binary dependency matrices encompassing data for hundreds of cancer cell lines can be downloaded from Project Score [28] and used with this function by calling CoRe.download_BinaryDepMatrix.

In order to identify CFGs using data from screening *N* cell lines, the Daisy Model introduced in [10] computes a fuzzy intersection of genes that are essential, i.e. fitness genes, in at least n* cell lines, where this number is defined a priori. ADaM generalizes this approach by (i) exploiting the bimodality of the distributions of the number of genes essential in each number of cell lines (Fig. 1A), and (ii) adaptively determining an optimal discriminative threshold of minimal number of cell lines n* that should be dependent on a given gene for calling that gene a CFG.

Briefly, for a binary matrix encompassing gene dependency profiles of *n* cell lines across thousands of screened genes, ADaM computes fuzzy intersections of genes *I*_*n*_, for each *n = 1, …, N*. These fuzzy intersections include genes with at least *n* dependent cell lines according to the input matrix. For each tested *n*, ADaM computes the true positive rate *TPR(n)* yielded by each *I*_*n*_ using the reference CFGs provided in input as positive controls. In parallel, ADaM also computes the number of genes that are expected to be essential in at least *n* cell lines by chance, via random permutations of the input matrix (Fig. 1B). Finally, ADaM determines the optimal n* as the largest value providing the trade-off between *TPR(n)* (inversely proportional to *n*) and the deviance of the number of genes with *n* dependent cell lines (directly proportional to *n*) from its expectation (Fig. 1C). The genes in the corresponding fuzzy intersection *I*_n*_ are predicted to be CFGs for the cell lines in the input dependency matrix.

As the distribution of genes that are CFGs in a specific number of tissue-lineage/cancer-types is also bimodal [11], this procedure can be executed in a two-step approach on large datasets of cancer dependency profiles, accounting for hundreds of cancer cell lines from multiple tissues, to predict pan-cancer CFGs (Fig. 1D). In the first step ADaM predicts tissue-lineage/cancer-type specific CFGs, then it iterates by adaptively determining the minimum number t* of tissue-lineages/cancer-types for which a gene should have been predicted as a specific CFG to be now predicted as a pan-cancer CFG. t* is determined by applying the same algorithm and criteria used to determine the n* across the tissue-lineages/cancer-types specific executions of ADaM (Fig. 1D). Particularly, this last operation is performed on a binary membership matrix with genes on the rows, tissue-lineages/cancer-types on the column and a 1 in position *[i, j]* indicating that the *i*-th gene is a CFG for *j*-th tissue-lineage/cancer-type.

All the functions called by CoRe.ADaM are exported and fully documented in the CoRe package. In addition, CoRe is equipped with the CoRe.PanCancer_ADaM wrapper function, implementing the two-step procedure to identify pan-cancer CFGs, and the CoRe.CS_ADaM function executing ADaM on a user-defined tissue-lineage/cancer-type, which can be used on dependency matrices from Project Score [28] and cell line annotations from the Cell Model Passports [32].

#### The Fitness Percentile method

The Fitness Percentile (FiPer) method works in an unsupervised manner. It identifies a set of common-essential genes (CEGs) by executing a single pooled analysis of data from multiple CRISPR-Cas9 screens. In addition, it takes as input a dependency matrix with quantitative fitness effect indicators of screened genes across cell lines.

We have designed and implemented in CoRe four novel variants of this method, all sharing the same initial step, which is executed for each individual gene in the input dependency matrix, in turn. In this step (i) all cell lines are sorted according to their dependency on the gene under consideration in decreasing order; (ii) the rank position of the gene under consideration resulting from sorting all screened genes according to their fitness effect is determined, for each screened cell line; (iii) a curve of the rank positions computed in (ii) is assembled considering the cell lines ordered as in (i): the fitness rank versus dependency percentile curve (FiPer curve, Fig. 1E).

It is reasonable to assume that genes involved in fundamental cellular processes (likely to be CEGs, such as RPL8 and RPL22 in Fig. 1E) will generally tend to rank amongst the most significant fitness genes for all the screened cell lines, including those that are the least dependent on them. This tendency can be extrapolated from the FiPer curves (thus measured in data coming from multiple CRISPR-Cas9 screens) and used to estimate the likelihood of a gene to be a CEG.

The CoRe.FiPer function implements four different methods to assess this tendency assigning a FiPer score to each gene differently. This is followed by a procedure that finally partitions all screened genes into two groups, with the first one containing the predicted CEGs.

The first method, the *Fixed* percentile (Fig. 1EF), considers as the FiPer score of a gene its fitness rank position in the cell line falling at the highest boundary of a very large dependency percentile of cell lines (90th by default). The *Average* method considers the average gene rank position in all the cell lines falling over a very large dependency percentile (90th by default). The *Slope* method fits a linear model onto each gene’s FiPer curve, then considers the slope of such a model as the gene FiPer score. In the final *AUC* method, the FiPer score of a gene is computed as the area under its FiPer curve.

Finally, a density function fitted onto the gene FiPer scores’ observed distribution (which is typically bimodal) using a kernel estimator and the score corresponding to the point of central local minimal density is used as a discriminative threshold to predict CEGs, which will be those with a FiPer score less than or equal to it (Fig. 1F).

CoRe includes also the CoRe.VisCFness function which visualises the tendency of a given gene to be a CEG within a dependency dataset provided in input and compares this tendency against that of a positive (RPL8 by default) and a negative (MAP2K1 by default) control, and producing the plots shown in Fig. 1E.

## Results

### Comparison with existing methods and state-of-the-art sets of core-fitness genes

**Fig. 2:**
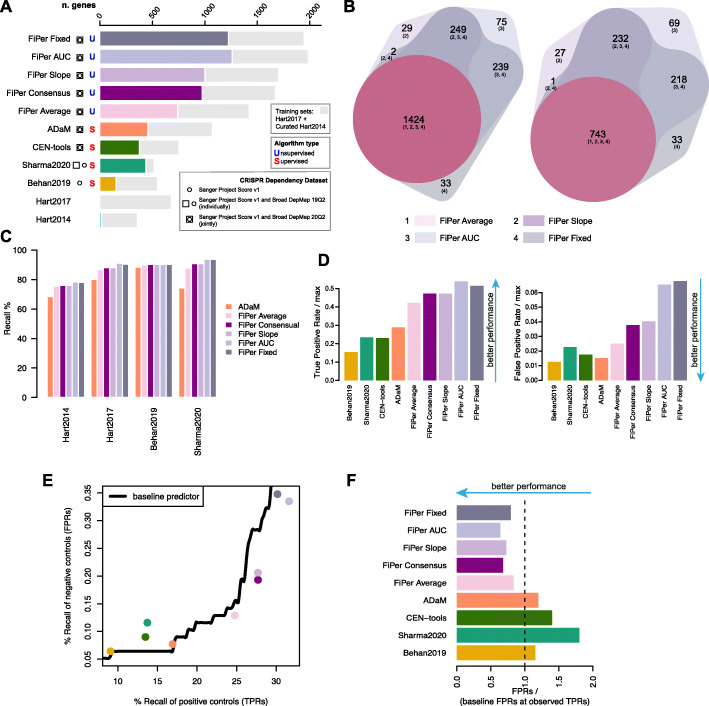
Comparison of CoRe output with state-of-the-art sets and methods. **A.** For each method predicting core-fitness essential genes (CFGs), common-essential genes (CEGs), or state-of-the-art (SOA) sets of CFGs, the overall length of the bar indicates the total number of genes, whereas the length of the coloured bar indicates the total number of predicted genes not included in any of the training sets. Squares/circles indicate the dataset analysed by each method or used to derive the considered SOA set, and letters indicate the nature of the method, i.e. (S)upervised or (U)nsupervised. **B**. Comparison of common-essential gene sets predicted by the four variants of the FiPer method (left) and considering novel hits only, i.e., excluding any gene belonging to any of the training sets (right). **C.** Recall of SOA sets of CFGs genes across CoRe methods’ predictions. **D.** True and False positive rates (TPRs/FPRs) of independent true/negative controls across SOA sets of CFGs, CoRe and other methods, relative to the maximal TPRs/FPRs attainable by the basal daisy model (DM) predictor of CFGs. **E.** Performance assessment accounting for set sizes. Each point corresponds to a different method or SOA set, with coordinates indicating their TPR/FPR, respectively along x- and y-axis. Black curve indicates the FPRs obtained by a baseline DM predictor at given TPRs. **F.** FPRs of all tested methods and SOA sets of CFGs relative to baseline performances. The length of each bar indicates the ratio between the FPR of the method or set under consideration and that of the baseline DM classifier at a TPR equal to that observed for the method or set under consideration

We compared the sets of CFGs and CEGs predicted by CoRe (through ADaM and all the FiPer variants) when applied to the largest integrative dataset of cancer dependency assembled to date, accounting for 17,486 genes and 855 cell lines from 30 different tissue-lineages and 43 cancer types (the DepMap dataset, Fig. 2AB) [19], with state-of-the-art sets of core-fitness genes derived from recent functional genetic screening datasets [10, 11, 30, 31]. We also included in the comparison the output of a logistic-regression based method, part of the recent CEN-tools software proposed in [30] applied to the DepMap dataset (Tables 1 and 2).

**Table 1:** State-of-the-art sets of core-fitness essential genes considered to benchmark CoRe

Set name	Set Type	Description and Source	Dataset of origin and method
*Hart2014*	State-of-the-art reference set of core-fitness essential genes	A set of 360 genes presented in [33] and used as a classification template by BAGEL: a supervised computational framework for quantifying gene essentiality significance in pooled library screens [10, 24].	Large collection of shRNA gene dependency profiles analysed with a linear algebra approach.
*Hart2017*	State-of-the-art reference set of core-fitness essential genes	A set of 684 genes introduced in [31].	BAGEL reanalysis of 17 genome-scale knockout screens in human cell lines performed with different libraries.
*Behan2019*	State-of-the-art reference set of core-fitness essential genes	A set of 553 genes presented in [11].	ADaM analysis of a large collection of gene dependency profiles from CRISPR-screens of 325 human cancer cell lines from different tissue-lineages/cancer-types (now part of the Project Score database [28]), using a manually curated version of the Hart2014 set (the *curated Hart2014* CFGs), as training. This was obtained by excluding from the Hart2014 set 34 genes, such as for example KRAS and CHD4, predicted to be cancer drivers by the intOGen pipeline [34, 35]
*Sharma2020*	State-of-the-art reference set of core-fitness essential genes	A set of 519 genes presented in [30].	Logistic regression approach (part of the CEN-tools software), which uses the BAGEL essential/never-essential genes as training sets, respectively the Hart2017 set and a set of 927 never-essential genes [10, 24]. This approach was individually applied to the dependency profiles from Project Score [28] and from the Broad DepMap portal [](https://depmap.org) (Release 19Q2). The final predicted set was composed of genes predicted as CFGs in the two analyses, excluding those in the training set. For the comparison with the unsupervised methods, this set was joined with the Hart2017 set (used in its training phase), rising up to 1182 genes.

**Table 2:** Sets of core-fitness and common-essential genes obtained by novel analyses of the DepMap dataset and considered to benchmark CoRe

Set name	Set Type	Number of genes	Dataset of origin
CEN-tools	Novel analysis	756 [For the comparison with the unsupervised methods, this set was joined with the curated Hart2014 set (used in its training phase), rising up to 1082 genes]	DepMap dataset [19].
CoRe ADaM	Novel analysis	1075	DepMap dataset [19]
CoRe FiPer average	Novel analysis	1424	DepMap dataset [19]
CoRe FiPer slope	Novel analysis	1704	DepMap dataset [19]
CoRe FiPer AUC	Novel analysis	1987	DepMap dataset [19]
CoRe FiPer Fixed	Novel analysis	1947	DepMap dataset [19]
CoRe FiPer consensus	Novel analysis	1673	DepMap dataset [19]

For the training phase of CEN-tools, we used the curated Hart2014 CFGs [11] (which we also used as reference set of positives while running ADaM), and the BAGEL never-essential genes [10], also curated as described in [11] (the curated BAGEL non-essential set).

In order to provide a fair benchmark with respect to sets outputted by the unsupervised methods, we also joined the Sharma2020 set, and the CEN-tools set with the reference CFGs used in their respective training phases, i.e., the Hart2017 set and the curated Hart2014 set. All the compared sets of CFGs and CEGs, the curated Hart2014 essential and curated BAGEL non-essential genes are included in Additional File 1**:** Table S1.

Amongst the predicted CFG sets derived from old and new executions of supervised methods, ADaM yielded the largest number of CFGs (460) not included in any of the training sets (curated Hart2014, Hart2017 and BAGEL non-essentials), when applied to the DepMap dataset (Fig. 2A). The Sharma2020 set ranked second (with 441), followed by the novel execution of CEN-tools (with 379) (Fig. 2A). As expected, all these sets, included more novel CFGs than Behan2019 (157 novel CFGs), likely due to its derivation from a sensibly smaller cancer dependency dataset (325 cell lines against 855 for ADaM and CEN-tools, and 325 + 489 for Sharma2020, Fig. 2A).

The 4 variants of the CoRe FiPer method yielded much larger and highly concordant sets of predicted CEGs (median = 1825.5, min = 1424 for FiPer average, max = 1987 for FiPer AUC, Fig. 2B), as well as novel hits (median = 1115, min = 743 for FiPer average, max = 1262 for FiPer AUC, Fig. 2B). The set of CEGs predicted by FiPer average was included in those predicted by all the other FiPer variants. For this reason, we decided to assemble a 5th FiPer set by intersecting the output of FiPer Slope, AUC and Fixed: the FiPer consensus set. This yielded 1673 genes, of which 975 were novel hits (Fig. 2A).

As a first exploratory analysis, we verified that all the sets of CFGs/CEGs outputted by the CoRe methods covered most of the state-of-the-art sets of CFGs (ADaM median Recall across prior known sets: 77.24%, FiPer median Recall across prior known sets, averaged across variants: 89.31%, Fig. 2C). Furthermore, while comparing overall CFG/CEG sets similarities, we observed three major clusters composed respectively by (i) the sets outputted by the FiPer variants, then (ii) Sharma2020, CEN-tools (both joined with respective training sets) and ADaM sets, and (iii) Hart2014, Hart2017 and Behan2019 sets (Additional File 2: Fig. S1). Taken together, these results suggest that the ADaM, CEN-tools and Sharma2020 sets might include similar numbers of novel CFGs, thus potentially extending in a similar way the other state-of-the-art CFG sets.

To investigate and compare true/false positives rates of the putative novel CFG/CEGs, we assembled, respectively, (i) a set of prior known CFGs (not included into any of the training sets) curated in [19, 21] using data from MsigDB [36], to be used as positive controls, and (ii) considered genes not expressed in human cancer cell lines (using data from the Cell Models Passports [32]) or whose essentiality is statistically associated with a molecular feature (thus very likely to be linked to specific molecular contexts) [19] as negative controls (Additional file 11: Additional methods and documentation, Additional File 3: Table S2). Both these sets are independent from the DepMap dataset.

Of the CFGs outputted by the supervised methods, ADaM had the best true positive rate (TPR), covering 29% of the positive controls screened in the DepMap. Sharma2020 ranked second (23.4%) followed by CEN-tools (23%) and Behan2019 (15%) (Fig. 2D). The median TPR for the FiPer variants was 47%, with FiPer AUC ranking first (54%) and FiPer Average last (42%). In terms of false positive rates (FPRs), Behan2019 performed the best, covering only 1.2% of the negative controls included in the DepMap dataset. ADaM ranked second (1.5%), followed by CEN-tools (1.7%) and Sharma2020 (2.3%). The median relative FPR for the FiPer variants was equal to 4% with FiPer average performing best (2.5%) and FiPer fixed worst (7%).

To account for differences in set sizes, which impact the observed TPRs/FPRs, we sought to compare the observed FPRs with those expected when using a baseline daisy model (DM) predictor of CFGs on the DepMap dataset, considering as the DM thresholds n* the *n* providing the observed TPRs of independent positive controls (Fig. 2E and Additional File 4: Fig. S2).

When considering the supervised methods, CoRe outperformed both CEN-tools and Sharma2020, yielding better ratios of FPRs with respect to those obtained at the observed TPRs by the DM (1.1 and 1.2 respectively for Behan2019 and ADaM, against 1.4 for CEN-tools and 1.8 for Sharma2020 (Fig. 2F)). Much better performances were obtained by the FiPer variants (median FPR / baseline ratio = 0.72) with FiPer AUC performing the best (0.64) and FiPer average the worst (0.83).

Optimal sets of CFGs/CEGs are expected to be essential in a vast majority of cancer cell lines: they have an average large negative impact on cellular fitness upon inactivation and are constitutively expressed in non-diseased tissues.

**Fig. 3:**
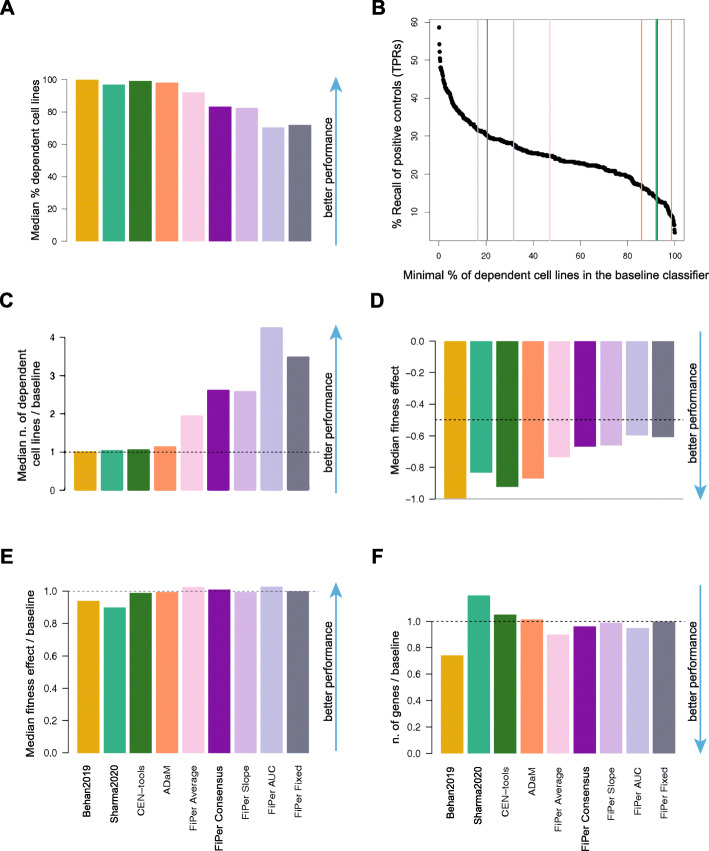
Fitness effects of CFG sets across cell lines. **A.** Median percentage of cell lines in which the genes in the predicted sets or core-fitness gene (CFG) or common-essential gene (CEG) sets are significantly essential. **B.** Threshold of minimal number of dependent cell lines *n* required by the baseline daisy model predictor (DM) to attain the true positive rates (TPRs) observed across tested methods. **C.** Ratios between median numbers of dependent cell lines for predicted sets divided by the threshold *n* of the DM to attain their TPRs. **D.** Median fitness effect exerted by the genes in the predicted CFG/CEG sets. **E.** Ratio between the median fitness effect in D and the median fitness effect exerted by the DM at the observed TPRs. **F.** Ratio between the number of genes in the predicted sets and those predicted by the DM at the observed TPRs

To evaluate these properties across the output of compared methods and state-of-the-art sets, we first measured the median number of cell lines dependent on the predicted sets of CFGs/CEGs (Fig. 3A). This was generally large for all the supervised methods, with the Behan2019 CFGs being essential (scaled fitness score < − 0.5, Additional file 11: Additional methods and documentation) in a median percentage of 99.8% cell lines of the DepMap dataset, followed by CEN-tools (98.9%), ADaM (98.1%) and Sharma2020 (96.8%). As expected, the CEGs yielded by the FiPer variants, were generally essential in smaller but still large percentages of cell lines (grand median = 82.3%, min = 70.2% for FiPer AUC - max = 92% for FiPer average). Nevertheless, when looking at the n* thresholds required by the baseline DM to attain the observed TPRs across predicted CFGs/CEGs (Fig. 3B), among the supervised methods the ADaM set showed again the best ratio between median number of dependent cell lines versus baseline (1.14, 98.1% against 86%), followed by CEN-tools (1.06, 98.9% against 93%), Sharma2020 (1.05, 96.8% against 92%) and Behan2019 (1.01, 99.8% against 98.6%) (Fig. 3C). The FiPer variants CEGs showed a median ratio between number of dependent cell lines versus DM thresholds at same TPR that was generally strikingly large across methods (median = 2.62, max 4.26 for FiPer AUC - min 1.95 for FiPer average).

The proximity to 1 of all the ratios for the supervised methods indicate that they all implicitly discover the DM’s optimal n*. ADaM goes further and selects a set of genes providing a TPR that would require a much lax minimal number of dependent cell lines to be achieved by the DM, thus resulting in an increased FPR. Furthermore, in these circumstances, the unsupervised methods massively outperform the supervised ones, showing the effectiveness of the FiPer criteria used to pick CEGs.

Next, we measured the median scaled fitness effect of the predicted CFGs/CEGs across cell lines, and we find it comfortably below − 0.8 -- i.e. 80% of the median effect for curated Hart2014 (Methods) -- for all the supervised methods (strongest effect = − 0.99 for Behan2019, weakest for Sharma2020 = − 0.83) and below − 0.5 -- i.e. half the fitness effect of the curated Hart2014 -- for the FiPer variants (strongest for FiPer average = − 0.73, weakest for FiPer AUC = − 0.59) (Fig. 3D).

Nevertheless, when comparing these values with their equivalent for the CFGs predicted by the baseline DM at the observed TPRs (excluding genes belonging to the training sets), ADaM was again the best performing supervised method (ratio between median fitness effect and baseline = 0.99), followed by CEN-tools (0.98), Behan2019 (0.93), and Sharma2020 (0.89). The median ratio for the FiPer variants was equal to 1.01 with FiPer AUC performing best (1.02) (Fig. 3E).

Finally, we found that all the compared methods predicted sets of CFGs/CEGs that were constitutively expressed in normal tissues at similar median levels (Additional File 5: Fig. S3). In addition, the CFG sets’ cardinality was systematically comparable or lower than that of CFG sets outputted by the baseline DM at the observed TPRs, with the exception of Sharma2020 and CEN-tools (Fig. 3F). Thus, these two sets were confirmed to be suboptimal and predicting larger numbers of CFGs with respect to the baseline DM but with worse FPRs at the observed TPRs (Fig. 2EF).

All these results were confirmed when the benchmark analyses were extended to the Hart2014 and Hart2017 sets, adding to CEN-tools and Sharma2020 their corresponding positive training sets and not excluding training set genes from positive/negative controls (thus considering 905 positive and 8040 negative controls - of which respective 466 and 695 are in the DepMap dataset) (Additional File 6: Fig. S4).

**Fig. 4:**
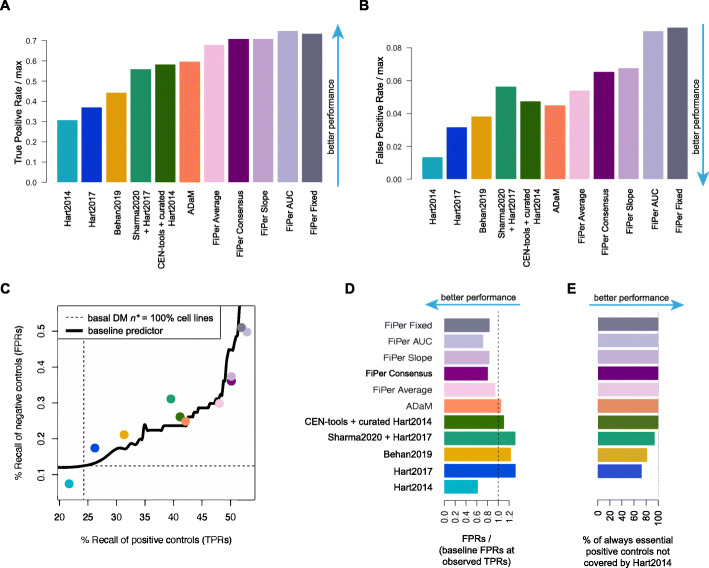
Performances of tested methods when accounting for genes in the training sets. **AB.** True and False positive rates (TPRs, FPRs) of independent true and negative controls across state-of-the-art (SOA) sets of core-fitness essential genes (CFGs), and sets outputted by CoRe and other methods, relative to the maximal TPRs/FPRs attainable by a basal daisy model (DM) predictor of CFGs. **C.** Performance assessment accounting for set size. Each point corresponds to a different method or SOA set, with coordinates indicating their TPR and FPR, respectively, along the x- and y-axis. The black curve indicates the FPRs obtained by a baseline DM predictor at given TPRs. **D.** FPRs of all tested methods and SOA sets of CFGs relative to baseline performances. The length of each bar indicates the ratio between the FPR of the set under consideration and that of the baseline DM classifier at a TPR equal to that observed for that set. **E.** Recall of positive control genes that are essential in 100% of the cell lines in the DepMap dataset and are not covered by the Hart2014 set, across all benchmarked sets

When considering all state-of-the-art sets of CFGs and supervised methods, we observed again that ADaM provides the best TPRs and FPRs (both absolute and relative to baseline, Fig. 4A-D).

The Hart2014 set showed the best FPRs versus baseline ratio, although this had to be extrapolated. In fact, this set had a TPR (21.7%) that was lower than that of the baseline DM classifier at the most stringent n* threshold (TPR = 24%, for 343 CFGs that are significantly essential in 100% of the screened cell lines) (Fig. 4C), and strikingly did not include 66 positive controls that are significantly essential in all the cell lines of the DepMap dataset (Fig. 4E). These 66 genes were all covered by all the methods executed on the DepMap dataset and only partially recalled by the Hart2017 (73%), the Behan2019 (82%) and the Sharma2020 (94%) sets.

**Fig. 5:**
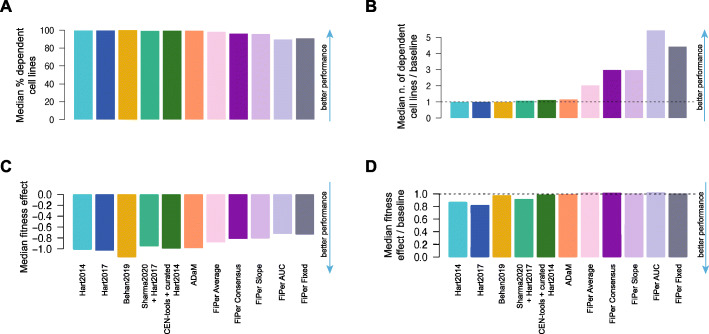
Comparison between CFG/CEG sets’ essentiality profile when accounting for genes in the training sets. **A.** Median percentage of cell lines in which the genes in the predicted sets or core-fitness gene (CFG) or common-essential gene (CEG) sets are significantly essential. **B.** Ratios between median numbers of dependent cell lines for predicted sets divided by the threshold *n* of the baseline daisy model predictor (DM) to attain their TPRs. **C.** Median fitness effect exerted by the genes in the predicted CFG/CEG sets. **D.** Ratio between the median fitness effect in D and the median fitness effect exerted by the DM at the observed TPRs

Taken together, these results strongly indicate that the CFGs derived from the DepMap dataset reliably extend state-of-the-art CFG sets and that, among those derived with supervised methods, the ADaM set is the most robust one. This was also confirmed in terms of number of cell lines dependent on the predicted CFGs (Fig. 5AB) and their median fitness effect (Fig. 5CD), relative to baseline performances.

### Methods’ performances using an independent cancer dependency dataset

We sought to compare the CFGs and CEGs outputted by the considered methods in terms of their median fitness effect across multiple screened models when using an independent cancer dependency dataset. To accomplish this, we considered an integrated dependency dataset generated by applying the DEMETER2 model to three large-scale RNAi screening datasets, covering 712 unique cancer cell lines [37], pre-processed as specified in the Additional file 11: Additional methods and documentation.

Also, in this case, the two versions of the ADaM CFGs sets outperformed the other supervised methods both in terms of absolute grand median fitness effect (− 0.79 and − 0.61, respectively, for Behan2019 and ADaM, versus − 0.6 and − 0.5, respectively for CEN-tools and Sharma2020) and ratio with respect to baseline DM (0.98 and 0.96, respectively for ADaM and Behan2019, versus 0.94 and 0.76, respectively for CEN-tools and Sharma2020, Additional File 7: Fig. S5). As we previously observed, the FiPer variants’ CEGs showed an overall milder grand median fitness effect (median = − 0.36) but much better ratios with respect to baseline (median = 0.99).

### Functional characterisation of predicted sets of core-fitness-essential and common-essential genes

**Fig. 6:**
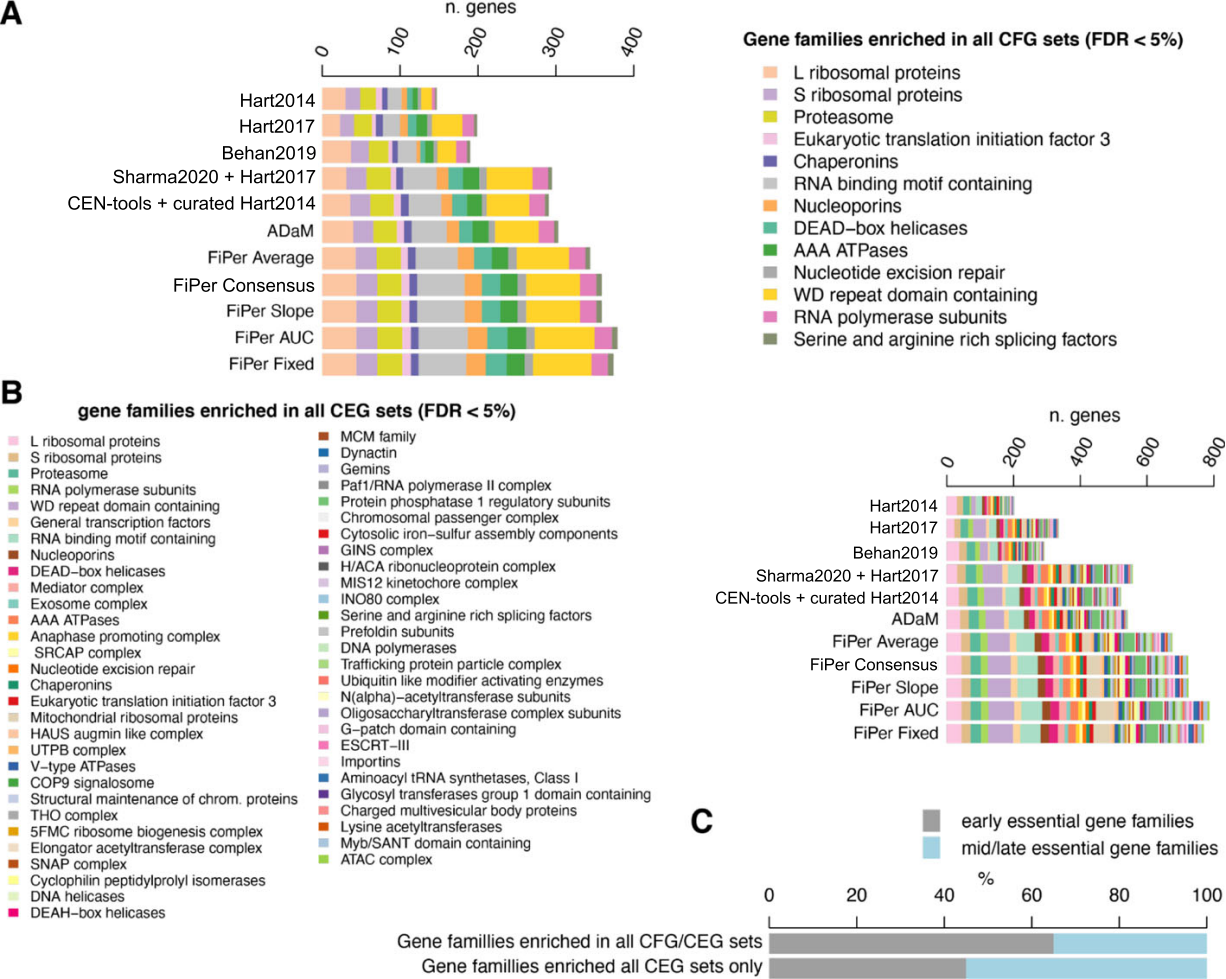
Functional characterisation of predicted core-fitness/common-essential genes. **A.** Gene families consistently significantly enriched (FDR < 5%) across all the state-of-the-art set of core-fitness essential genes (CFGs) and those outputted by the supervised methods. **B.** Gene families consistently and significantly enriched (FDR < 5%) across all the common-essential gene (CEG) sets outputted by the CoRe FiPer variants. **C** Percentage of early and mid/late essential gene families that are also always enriched across CFG and CEG sets or in CEG sets only

We performed a systematic statistical enrichment analysis of gene families across all sets of CFGs and CEGs considered in our benchmark, to functionally characterise them. This yielded a set of 13 families significantly enriched (FDR < 5%) consistently across all the state-of-the-art sets of CFGs as well as in the CFGs outputted by all tested supervised methods (Fig. 6A and Additional File 8: Table S3), thus worthy to be considered as bonafide true positive enrichments in human core-fitness essential genes (the core-fitness families). These families encompass most of the true positive controls used in our benchmark (ribosomal protein genes, proteasome, RNA polymerase [36]), as well as other plausible families, such as proteins involved in the initiation phase of eukaryotic translation [38], chaperonins [39], nucleoporins [40, 41] and less immediate hits, such as AAA-ATPase [42, 43] and WD repeat domain families [44, 45].

The coverage of these families was much larger for the more recent CFG sets when compared to the state-of-the-art CFGs, with ADaM and Sharma2020 performing best (average Recall across families = 57 and 54%, respectively). The unsupervised methods further extended the coverage of these gene families with average Recalls ranging from 63% (for FiPer average) to 68% (for FiPer AUC), with a median of 65%.

57 gene families were significantly enriched (FDR < 5%) consistently across the CEG sets outputted by the FiPer methods (Fig. 6B). These included all the 13 core-fitness families plus 44 additional groups (the common-essential families) such as COP9 signalosome [46, 47], mediator complex [48], SNAP complex [49, 50] and prefoldin subunits [51], to name a few.

When comparing the predicted CFG and CEG sets with the gene-essentiality timing characterisation presented in [52], we observed in the former more genes exerting a negative fitness effect at an early time point upon knock-out (early-essential genes), whereas the latter included more families enriched in genes whose effect on fitness can be detected only at a later time point (late-essential genes) (Fig. 6C), such as exosome complex [53], dynactin [54] and ubiquitin-like modifier activating enzymes [55, 56].

### Evaluation of core-fitness gene sets as template predictors of cell line specific essential genes

We performed a final analysis evaluating each state-of-the-art set of core-fitness essential genes (CFGs), and those outputted by CEN-tools and ADaM when applied to the DepMap dataset, as a template classifier of cell line specific essential genes with BAGEL: a widely used bayesian method to estimate gene essentiality significance in pooled CRISPR-cas9 screens [24].

To this aim, we analysed with BAGEL the dependency profiles in the DepMap dataset generated at Sanger, and preprocessed with CRISPRcleanR [21] (Additional file 11: Additional methods and documentation), obtaining 7 instances of BAGEL Bayes Factor (BF) matrices, quantifying the likelihood of each gene to be essential in each cell line, using each of the benchmarked set in turn as positive reference set of essential genes in the BAGEL classification template. To evaluate the robustness of the obtained cell line specific BFs we assembled sets of cell line specific positive/negative essential-gene controls.

As positive control, we considered putative oncogenetic dependencies arising from oncogenes (from [35]) found mutated or copy number amplified in a cell line (using data from the Cell Model Passports [32]), whereas wild-type and non-expressed (FPKM < 0.1) oncogenes were considered as negative controls (Additional File 9: Table S4).

**Fig. 7:**
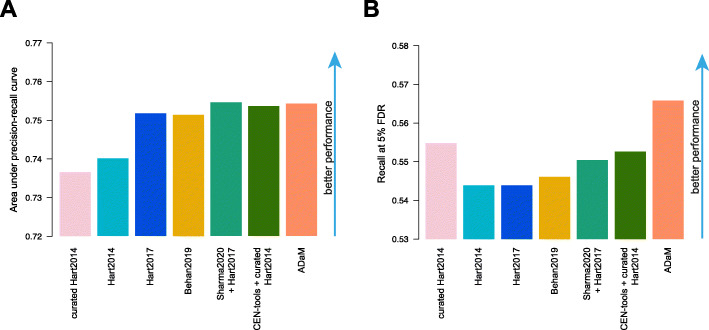
Performances of the benchmarked sets as template classifiers of cell line specific essential genes. **A.** Area under precision-recall curve obtained when predicting cell line specific oncogenetic addictions versus not expressed oncogenes with rank-based classifiers yielded by gene essentiality Bayesian factors. These are computed by BAGEL using each of the benchmarked sets as positive classification template. **B.** Recall of cell line specific oncogenetic addictions at 5% FDR of not expressed oncogenes yielded by each benchmarked set when used as for A

Then, we assessed the 7 BF matrices, pooling all included values together and considering them as a unique rank-based predictor (the larger the BF the higher the likelihood of a gene to be essential) of cell line specific essential genes, by means of receiver operating characteristic (ROC) analyses (Additional file 11: Additional methods and documentation). Particularly, for each benchmarked set we computed the area under the BF-rank induced precision-recall curve (AUPRC) (Fig. 7A and Additional File 10: Fig. S6) and the recall of positive controls at 5% FDR (Fig. 7B). All the sets of CFGs outputted by CEN-tools and CoRe applied to the DepMap dataset (Table 2) outperformed the state-of-the-art sets of CFGs, showing a better ability to detect as significantly essential mutated oncogenes, when used as a template for BAGEL. Above all, ADaM achieved the highest recall at 5% FDR (Additional file 11: Additional methods and documentation).

### Computational efficiency

We measured and compared running times of the benchmarked methods applied to the DepMap dataset, on different operating systems as well as on Google CoLab, a Jupyter notebook service hosted by Google servers (Table 3). The CoRe FiPer methods were between 16 (FiPer slope vs ADaM on Ubuntu 16.04 LTS) to 98 (FiPer fixed vs ADaM on CoLab) times faster than ADaM and between 31 (FiPer slope vs CEN-tools on Ubuntu 16.04 LTS) to 123 times (FiPer fixed vs CEN-tools on CoLab) faster than CEN-tools. Across FiPer variants, the slope one was the slowest, probably due to fitting of a linear regression model to a discrete distribution of gene fitness-rank-positions. Nevertheless, FiPer’s running time was still significantly lower than ADaM and both outperformed CEN-tools, which was the method with the longest running times, invariantly across operating systems.

**Table 3:** Computational efficiency across methods. Assessments of running time of the six compared methods when executed on different operating systems and on Google Colab

Algorithm	MacOS Big Sur	Ubuntu 16.04 LTS	Windows 10	CoLab (Ubuntu 18.04 LTS)
ADaM	7 mins 38.23 s	6 mins 30.88 s	10 mins 36.56 s	12 mins 19.95 s
CEN-tools	10 mins 22.76 s	12 mins 34.74 s	15 mins 24.33 s	15 mins 32.69 s
FiPer (average)	4.93 s	6.52 s	6.67 s	8.26 s
FiPer (AUC)	5.77 s	7.54 s	7.83 s	9.79 s
FiPer (fixed)	4.78 s	5.55 s	5.76 s	7.57 s
FiPer (slope)	18.97 s	24.07 s	29.39 s	32.26 s

## Discussion

We introduced CoRe: an open-source R package implementing both existing and novel methods for the identification of core-fitness essential genes (CFGs) --at two different levels of stringency-- from joint analyses of multiple CRISPR-Cas9 pooled recessive screens. We robustly and extensively benchmarked CoRe against state-of-the-art sets of core-fitness genes and other CFGs discovery methods, using the largest integrative dataset of cancer dependencies to date. We observed that the sets of core-fitness essential and common-essential genes (CEGs, outputted by the less stringent methods) predicted by CoRe are much more comprehensive and robust, in terms of true and false positive rates (TPRs, FPRs) both absolute and relative to a baseline classifier. For the latter, we considered a simple baseline daisy model (DM) model [10] outputting as CFGs those genes exerting a negative effect on fitness upon CRISPR-Cas9 targeting in at least an optimal minimal number of screened models, which is known a priori. We also demonstrated that both CoRe and other methods can implicitly detect this optimal DM threshold, with the CoRe methods going much further and accurately predicting sets of genes that are essential in numbers of cell lines that are larger than this threshold. This is much more evident for the less stringent methods implemented in CoRe (i.e., the FiPer variants), thus showing the effectiveness of their underlying algorithm (based on genes’ fitness percentile curves), which selectively picks likely true CEGs. Particularly, across these variants, the FiPer AUC method performs the best even when compared to a consensus set of CEGs obtained by intersecting the output of all the other FiPer variants. Consistently, AUC is the FiPer variant implemented/executed by default by CoRe. However, the other variants are also implemented in CoRe and can be executed for reproducibility purposes.

Contrary to other methods, the sets of CFG/CEG predicted by CoRe are also smaller than those outputted by a baseline DM predictor attaining the same true positive rates, and our benchmark results were all confirmed when extending the analysis to gene sets used in the training phase of at least one of the compared methods, and when considering an independent RNAi based cancer dependency dataset.

Furthermore, we found that the CoRe CFGs/CEGs extend gene families covered by previous state-of-the art sets and methods, with the FiPer methods being able to detect more subtle yet consistent fitness effects and core late essential genes. Finally, the CoRe CFGs/CEGs are all constitutively expressed in non-diseased tissue, pointing to the primary role which these genes play inside the cell. Indeed, it has been shown that higher essentiality is correlated with higher expression and association in important biological pathways [57].

Importantly, our final benchmark analysis also suggests that the CFGs yielded by our novel analyses of the DepMap dataset might be better suited than the reference positive control sets currently used [31, 33] as positive predictor template when estimating cell line specific essential genes with a supervised classification method, such as BAGEL [24].

The development of new tools exploiting the wealth of data currently being generated from CRISPR screens is of paramount importance [58]. Paired with the generation of new data from large efforts and collaborative endeavours, such as for example the Cancer Dependency Map [29, 59], this will be vital for identifying new oncology therapeutic targets, as well as for the characterisation of novel human core essential genes. Nevertheless, another key need is to couple CRISPR screening data with other genetic and molecular information of the screened models and data from ‘normal’ samples. A major reason for this is that a context-specific essential gene in a given cancer genetic background might be, for example, too toxic if suppressed in vivo or, in the opposite case, a gene characterized by a pan-essentiality profile in cancer might show reduced on-target toxicities [60].

## Conclusions

The identification of core-fitness genes has important implications in different areas of the life sciences: from drug discovery and cancer therapy to the study of genetic networks. However, different strategies are required according to the type of biological question being investigated. From this perspective, the utility of CoRe is twofold. In fact, when performing functional genetic studies or aiming at identifying novel CFGs, we recommend adopting a more stringent approach, such as ADaM, which can guarantee higher confidence. On the other hand, when the focus is on the identification of new therapeutic targets, thus, to seek new promising context-specific essential genes, the opposite is true. Therefore, applying a less stringent algorithm, such as the FiPer method (particularly the FiPer AUC) allows a larger number of genes to be classified as common-essentials, thus ruling out confounding genes that may skew the outcome of the analysis.

In addition, the CoRe workflow can be adapted to users’ needs and contingencies and it is compatible with many pre-processing methods and tools to estimate fitness effect significance. For example, the recently introduced Chronos tool [61] (accounting for cell population dynamics while estimating gene essentiality) could be used instead of CERES [12]. In addition, when copy number alteration profiles are not available for the screened models, the unsupervised method CRISPRcleanR [21] could be used to correct for gene-independent responses to CRISPR-Cas9 targeting. Furthermore the recent BAGEL2 tool [62] can be used in the initial binarization of essentiality scores, required for ADaM.

Finally, where sufficient data is available, i.e. enough screened models, the algorithms implemented in CoRe could be used to analyze specific subsets of cancer cell lines hosting certain molecular features (e.g. KRAS mutations in colorectal carcinoma), allowing identifying/comparing subtype specific core-fitness genes, which would be of particular interest for translational cancer research.

With the increasing availability of comprehensive cancer dependency maps [29], tools such CoRe will be arguably more and more needed in the future, and they will contribute translating data and findings from such efforts into novel therapeutic target candidates.

### Availability and requirements

Project name: CoRe.

Project home page: https://github.com/DepMap-Analytics/CoRe

Operating system(s): Platform independent.

Programming language: R, python.

Other requirements: R 3.5.0 or higher, python 3 or higher.

License: GNU GPLv3.

Any restrictions to use by non-academics: None.

## Supplementary information

**Additional File 1: Table S1.** All compared sets of core-fitness and common-essential genes with annotations.

**Additional File 2: Fig. S1.** Core-fitness essential and common-essential (CFG, and CEG) sets similarity. **A.** Heatmap showing core-fitness set membership for all genes predicted as core-fitness (in the columns) by at least one method/set. **B.** Jaccard coefficient of similarity among compared core-fitness sets. The Jaccard similarity is defined as the size of the intersection divided by the size of the union of two sets.

**Additional File 3: Table S2.** Positive and negative control genes and their membership to training sets and DepMap datasets.

**Additional File 4: Fig. S2.** Baseline daisy model predictor (DM) performances on the DepMap dataset. **A.** Number of genes predicted as core-fitness by a baseline DM classifier (baseline core-fitness genes (CFGs)), as a function of the minimal required number of dependent cell lines, respectively y and x axis. **B.** Recall of positive controls (TPR) for each set of baseline core-fitness genes (CFGs), across all possible minimal numbers of dependent cell lines (baseline TPRs). **C.** Recall of negative controls (FPR) for each set of baseline core-fitness genes (CFGs), across all possible minimal numbers of dependent cell lines (baseline FPRs). **D.** Baseline FPR as a function of baseline TPR.

**Additional File 5: Fig. S3.** Basal expression level of predicted CFG/CEG sets in normal tissues, in terms of Fragments Per Kilobase of transcript per Million mapped reads (FPKM) extracted from the Genotype-Tissue Expression (GTEx) portal database.

**Additional File 6: Fig. S4.** Baseline DM performances on the DepMap dataset when including genes in the training sets. **A.** Recall of positive controls (TPR) for each set of baseline core-fitness genes (CFGs) predicted by the DM as a function of the minimal required number of dependent cell lines, respectively y and x axis, across all possible minimal numbers of dependent cell lines (baseline TPRs). **B.** Recall of negative controls (FPR) for each set of baseline core-fitness genes (CFGs), across all possible minimal numbers of dependent cell lines values (baseline FPRs). **C.** Baseline FPR as a function of baseline TPR.

**Additional File 7: Fig. S5.** Performances’ comparison considering an independent cancer dependency dataset. **A.** Fitness effect exerted by the predicted core-fitness/common-essential gene (CFG/CEG) sets using an independent RNAi based cancer dependency dataset. **B.** Ratio between the median fitness effect of each CFG set divided by the median fitness effect exerted by the baseline daisy model predictor at the observed TPRs.

**Additional File 8: Table S3.** Gene family enrichment analysis results.

**Additional File 9: Table S4.** Cell line specific oncogenetic addictions, i.e. point mutated or copy number amplified oncogenes (1) and not expressed oncogenes (− 1).

**Additional File 10: Fig. S6.** Precision-recall curves of oncogene addictions versus not-expressed oncogenes yielded by rank-based classifiers based on bayesian factors computed with BAGEL when using the compared sets of CFGs as positive training sets.

**Additional File 11:** Additional methods and documentation.

## Data Availability

CoRe is publicly available as an open-source platform independent R package at https://github.com/DepMap-Analytics/CoRe (10.5281/zenodo.5603296, license: GPL (> = 3)). An interactive vignette, with demonstrations and examples is available at https://rpubs.com/AleVin1995/CoRe. The package includes built-in visualisation and benchmarking functions and their related data objects. It also contains interface functions for downloading and processing state-of-the-art cancer dependency datasets from Project Score [28], as well as updated cancer cell line annotations from the Cell Models Passports [32]. Finally, results from benchmarking CoRe against state-of-the-art sets of CFGs and other CFGs identification methods, with corresponding figures, are fully reproducible executing the Jupyter notebook (also compatible with Google CoLab) available at: https://github.com/DepMap-Analytics/CoRe/blob/master/notebooks/CoRe_Benchmarking.ipynb. The latest version of the integrated Sanger and Broad essentiality matrix processed with CERES [12] is available on the DepMap portal (https://www.depmap.org/broad-sanger/integrated_Sanger_Broad_essentiality_matrices_20201201.zip). The cancer cell lines annotation file is available on the Cell Model Passport (annotation file version 20210326, https://cog.sanger.ac.uk/cmp/download/model_list_20210326.csv.gz). The CEN-tools package [30] is available at https://gitlab.ebi.ac.uk/petsalakilab/centools/-/tree/master/CEN-tools. The BAGEL2 software is available at https://github.com/hart-lab/bagel. The DEMETER v6 04/20, an dataset is available at https://ndownloader.figshare.com/files/11489669. The release 1 of the Sanger cancer dependency dataset is available at https://score.depmap.sanger.ac.uk/downloads. All the remaining data are embedded as native R objects in the CoRe package.

## Abbreviations

CRISPR: Clustered Regularly Interspaced Short Palindromic Repeats
sgRNA: Single Guide RNA
CFG: Core Fitness Gene
CEG: Common Essential Gene
DM: Ddaisy Model
ADaM: Adaptive Daisy Model
FiPer: Fitness Percentile
DepMap: Dependency Map
AUC: Area Under the Curve
TPR: True Positive Rate
FPR: False Positive Rate
FPKM: Fragments Per Kilobase of transcript per Million mapped reads
BF: Bayes Factor
ROC: Receiver Operating Characteristic
AUPRC: Area Under the PRecision / Recall curve
FDR: False Discovery Rate
