# Supplementary figures and images for: CoRe: a robustly benchmarked R package for identifying core-fitness genes in genome-wide pooled CRISPR-Cas9 screens
Source: BMC Genomics. 2021 Nov 17;22:828. doi: 10.1186/s12864-021-08129-5 (PMC8597285; doi:10.1186/s12864-021-08129-5)

**A**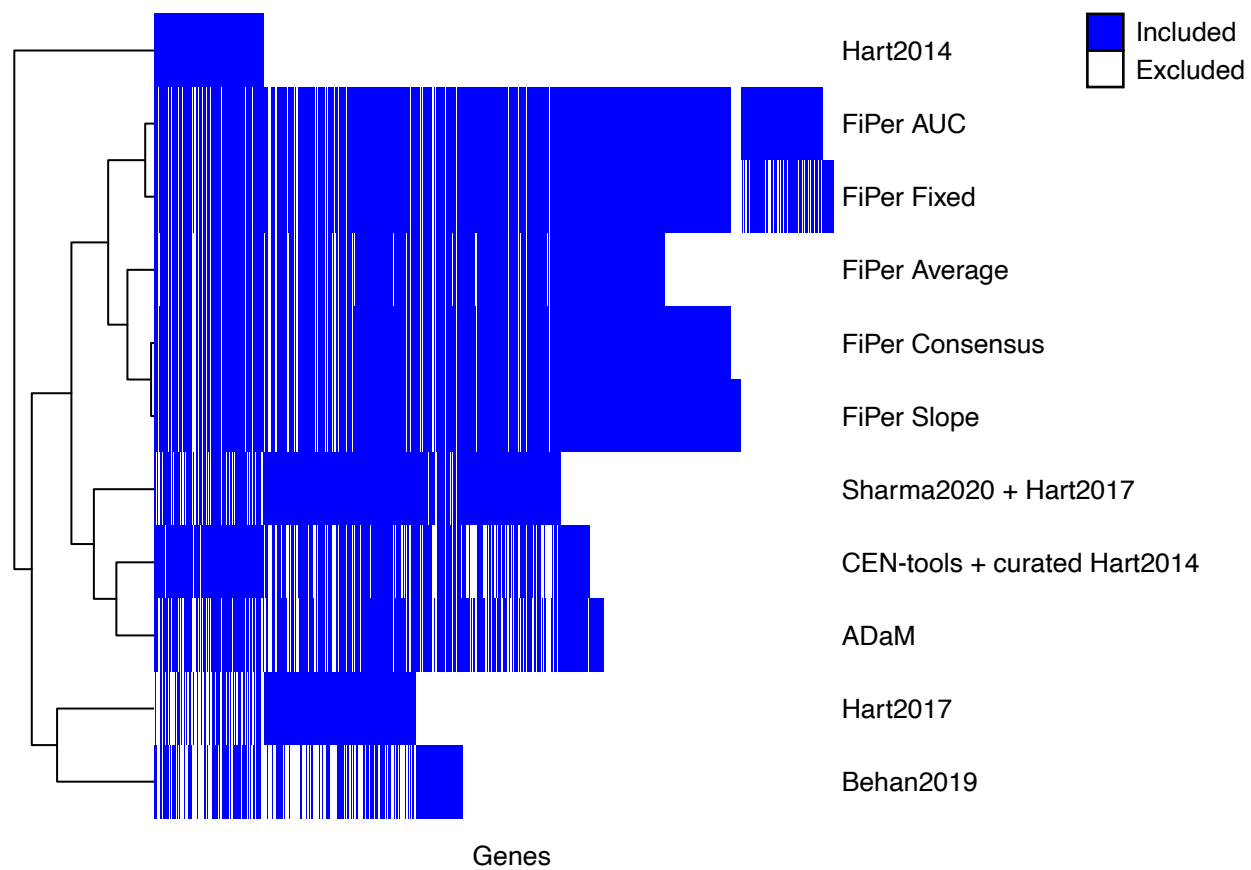**B**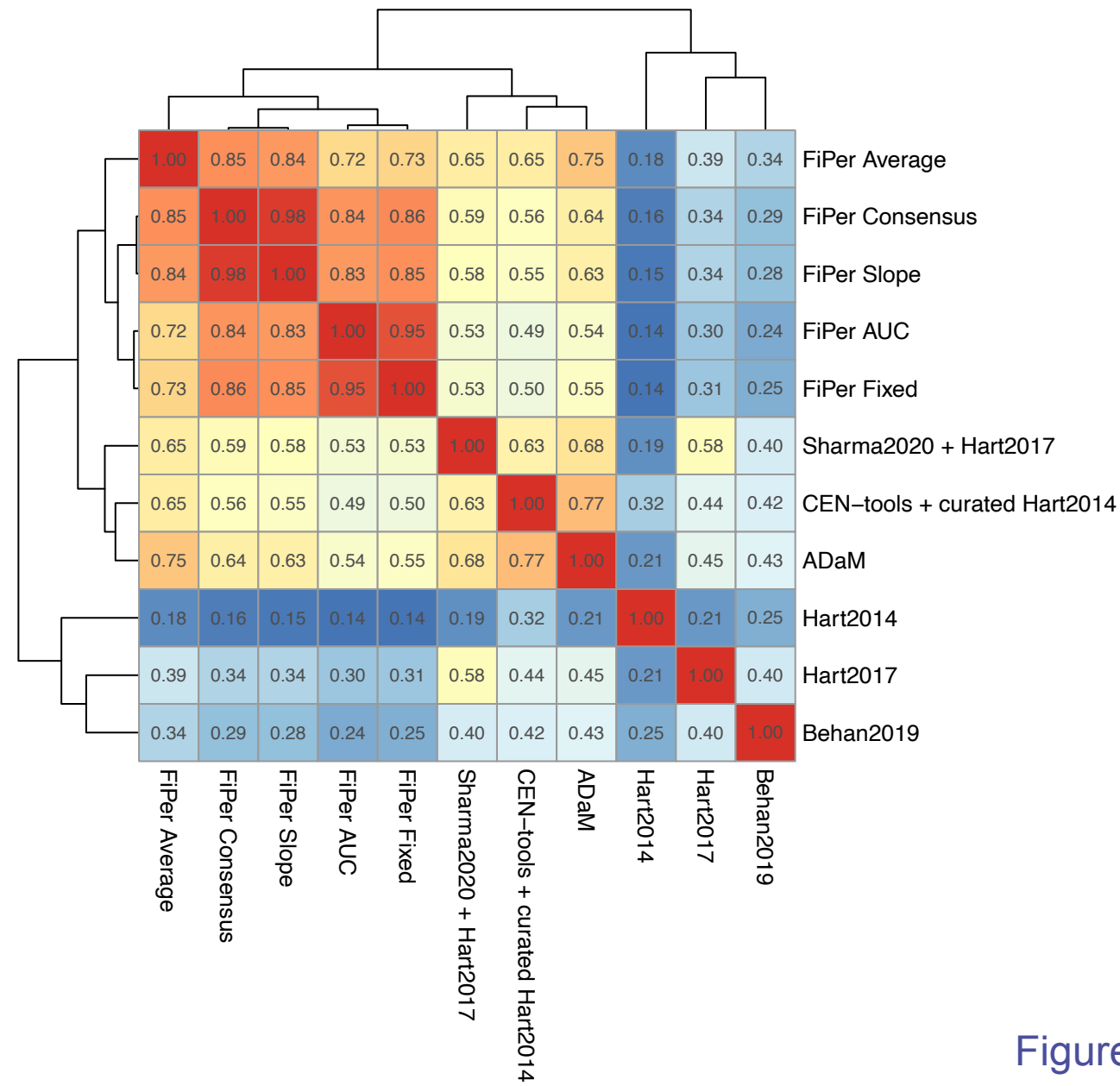

Figure S1

Supplement: Supplementary file 2 — Additional File 2: Fig. S1. Core-fitness essential and common-essential (CFG, and CEG) sets similarity. A. Heatmap showing core-fitness set membership for all genes predicted as core-fitness (in the columns) by at least one method/set. B. Jaccard coefficient of similarity among compared core-fitness sets. The Jaccard similarity is defined as the size of the intersection divided by the size of the union of two sets. [file 12864_2021_8129_MOESM2_ESM.pdf]

**A**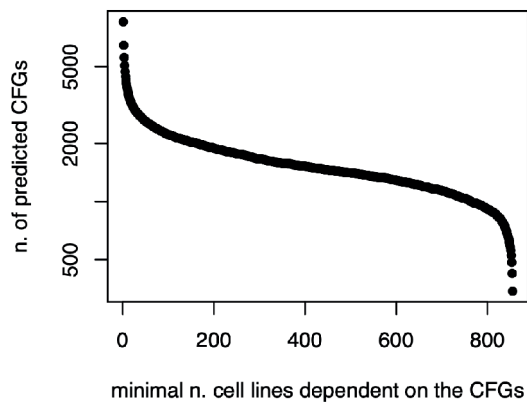**B**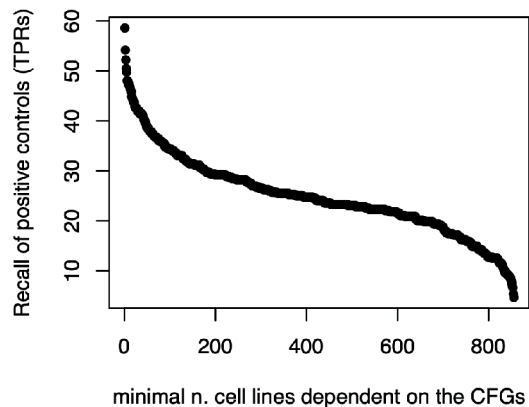**C**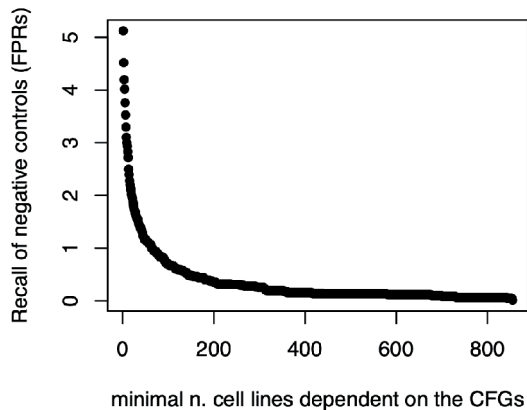**D**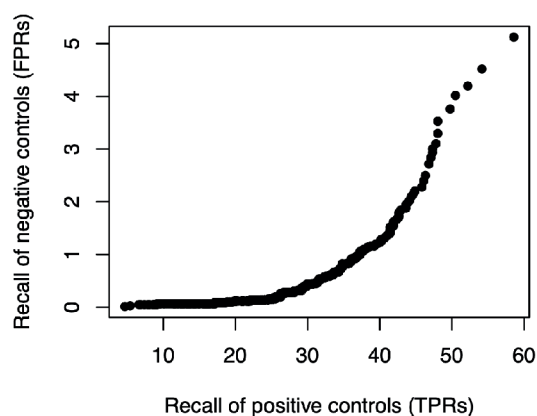

Supplement: Supplementary file 4 — Additional File 4: Fig. S2. Baseline daisy model predictor (DM) performances on the DepMap dataset. A. Number of genes predicted as core-fitness by a baseline DM classifier (baseline core-fitness genes (CFGs)), as a function of the minimal required number of dependent cell lines, respectively y and x axis. B. Recall of positive controls (TPR) for each set of baseline core-fitness genes (CFGs), across all possible minimal numbers of dependent cell lines (baseline TPRs). C. Recall of negative controls (FPR) for each set of baseline core-fitness genes (CFGs), across all possible minimal numbers of dependent cell lines (baseline FPRs). D. Baseline FPR as a function of baseline TPR. [file 12864_2021_8129_MOESM4_ESM.pdf]

## basal expression in normal tissues

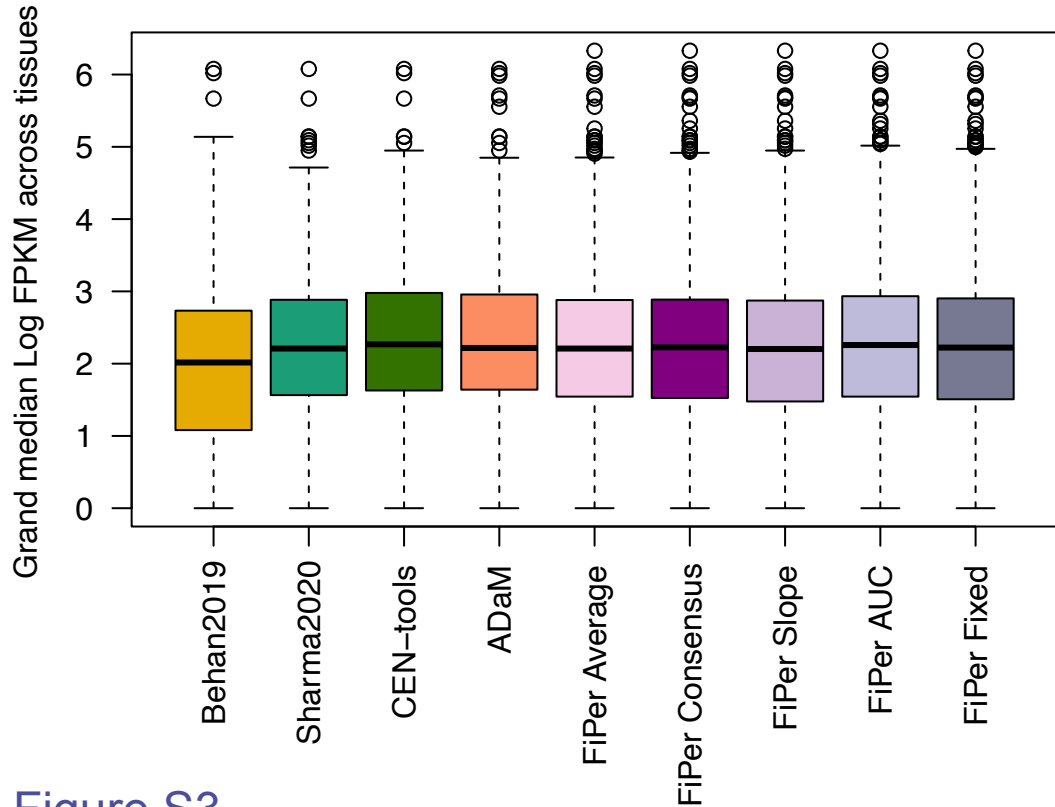

Figure S3

Supplement: Supplementary file 5 — Additional File 5: Fig. S3. Basal expression level of predicted CFG/CEG sets in normal tissues, in terms of Fragments Per Kilobase of transcript per Million mapped reads (FPKM) extracted from the Genotype-Tissue Expression (GTEx) portal database. [file 12864_2021_8129_MOESM5_ESM.pdf]

**A**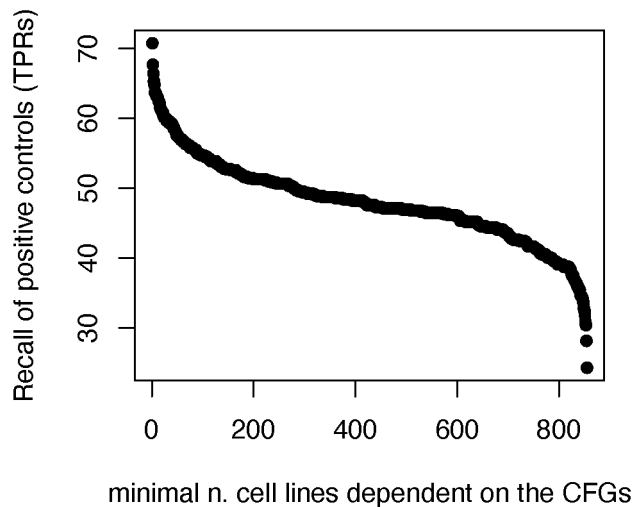**B**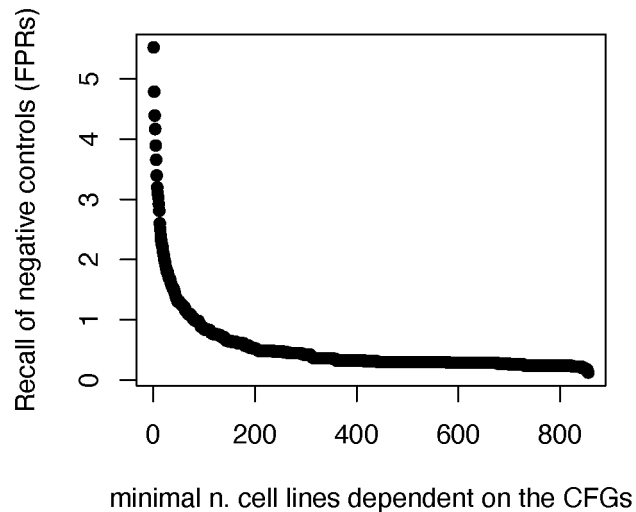**C**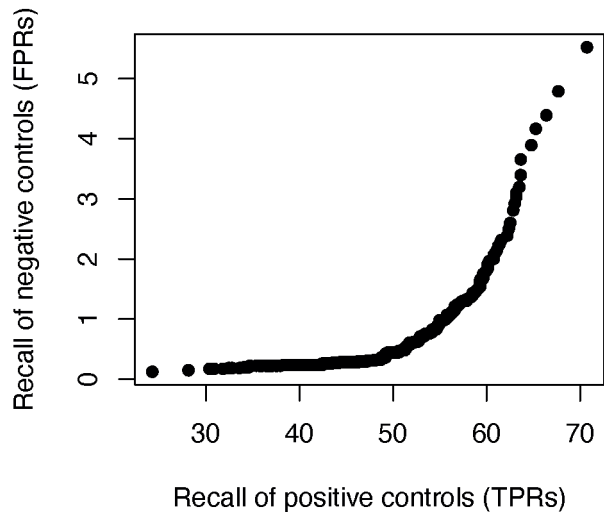

Figure S4

Supplement: Supplementary file 6 — Additional File 6: Fig. S4. Baseline DM performances on the DepMap dataset when including genes in the training sets. A. Recall of positive controls (TPR) for each set of baseline core-fitness genes (CFGs) predicted by the DM as a function of the minimal required number of dependent cell lines, respectively y and x axis, across all possible minimal numbers of dependent cell lines (baseline TPRs). B. Recall of negative controls (FPR) for each set of baseline core-fitness genes (CFGs), across all possible minimal numbers of dependent cell lines values (baseline FPRs). C. Baseline FPR as a function of baseline TPR. [file 12864_2021_8129_MOESM6_ESM.pdf]

**A**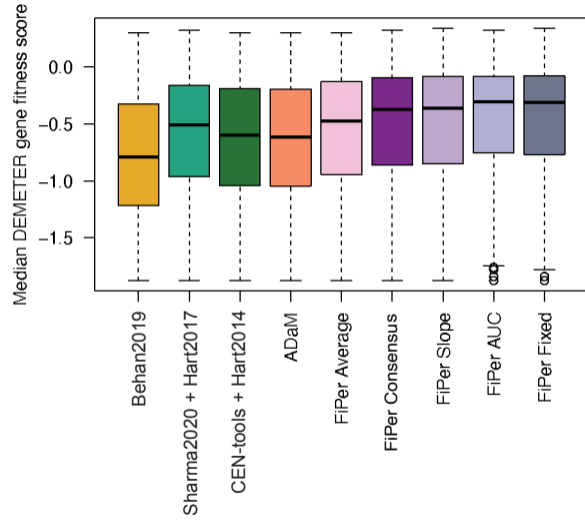**B**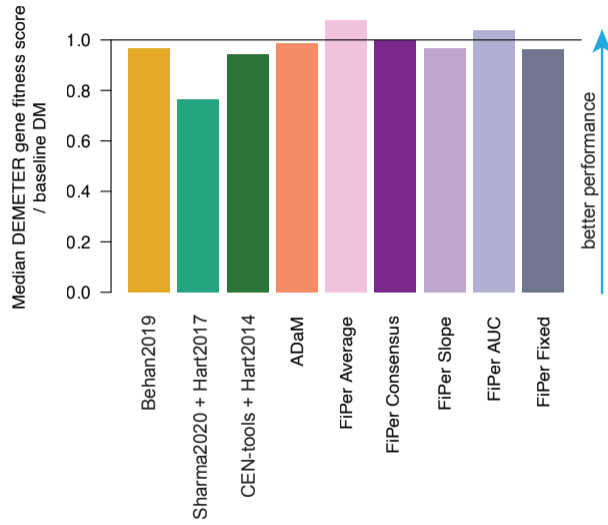

Figure S5

Supplement: Supplementary file 7 — Additional File 7: Fig. S5. Performances’ comparison considering an independent cancer dependency dataset. A. Fitness effect exerted by the predicted core-fitness/common-essential gene (CFG/CEG) sets using an independent RNAi based cancer dependency dataset. B. Ratio between the median fitness effect of each CFG set divided by the median fitness effect exerted by the baseline daisy model predictor at the observed TPRs. [file 12864_2021_8129_MOESM7_ESM.pdf]

**ADaM**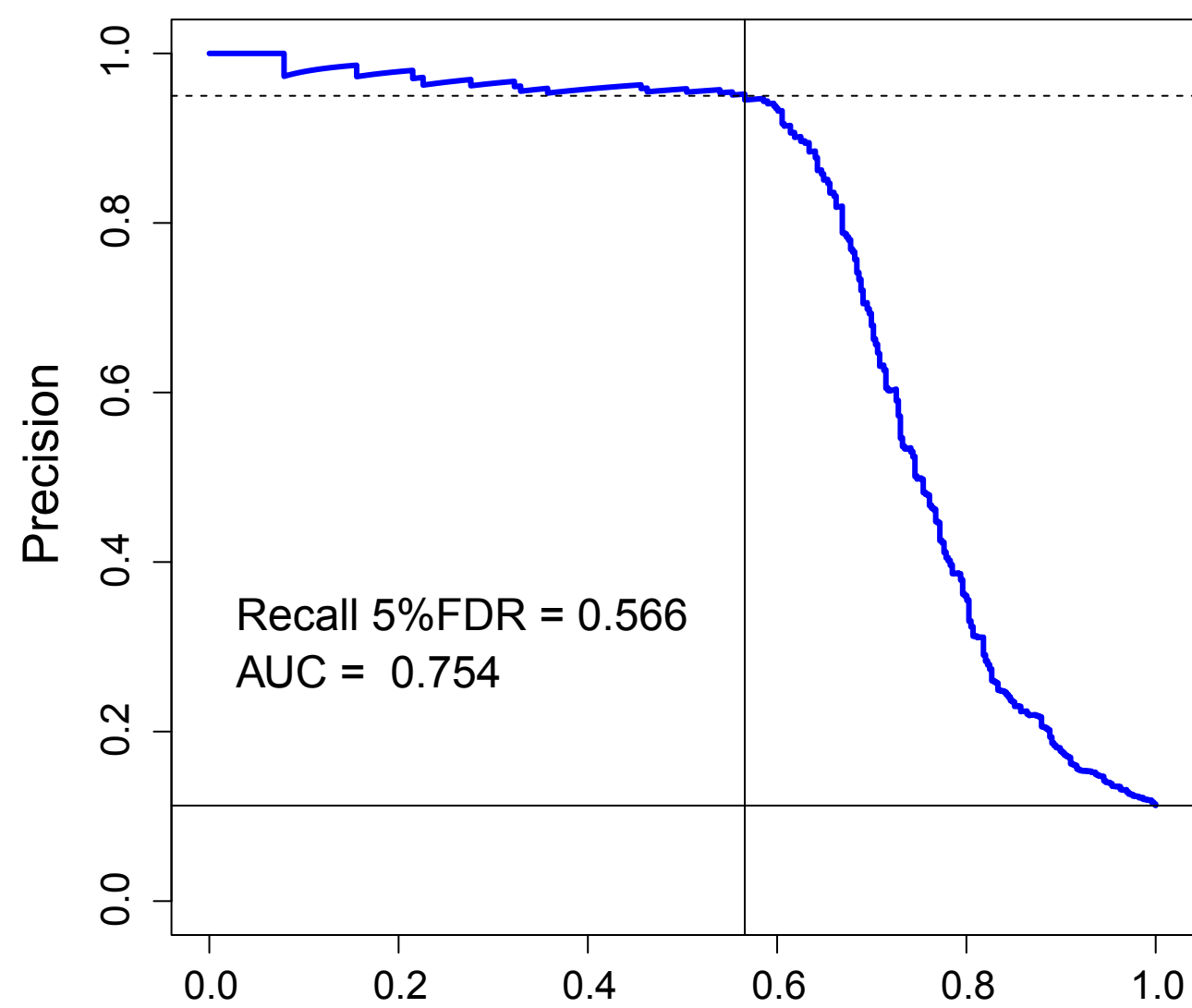**Behan2019**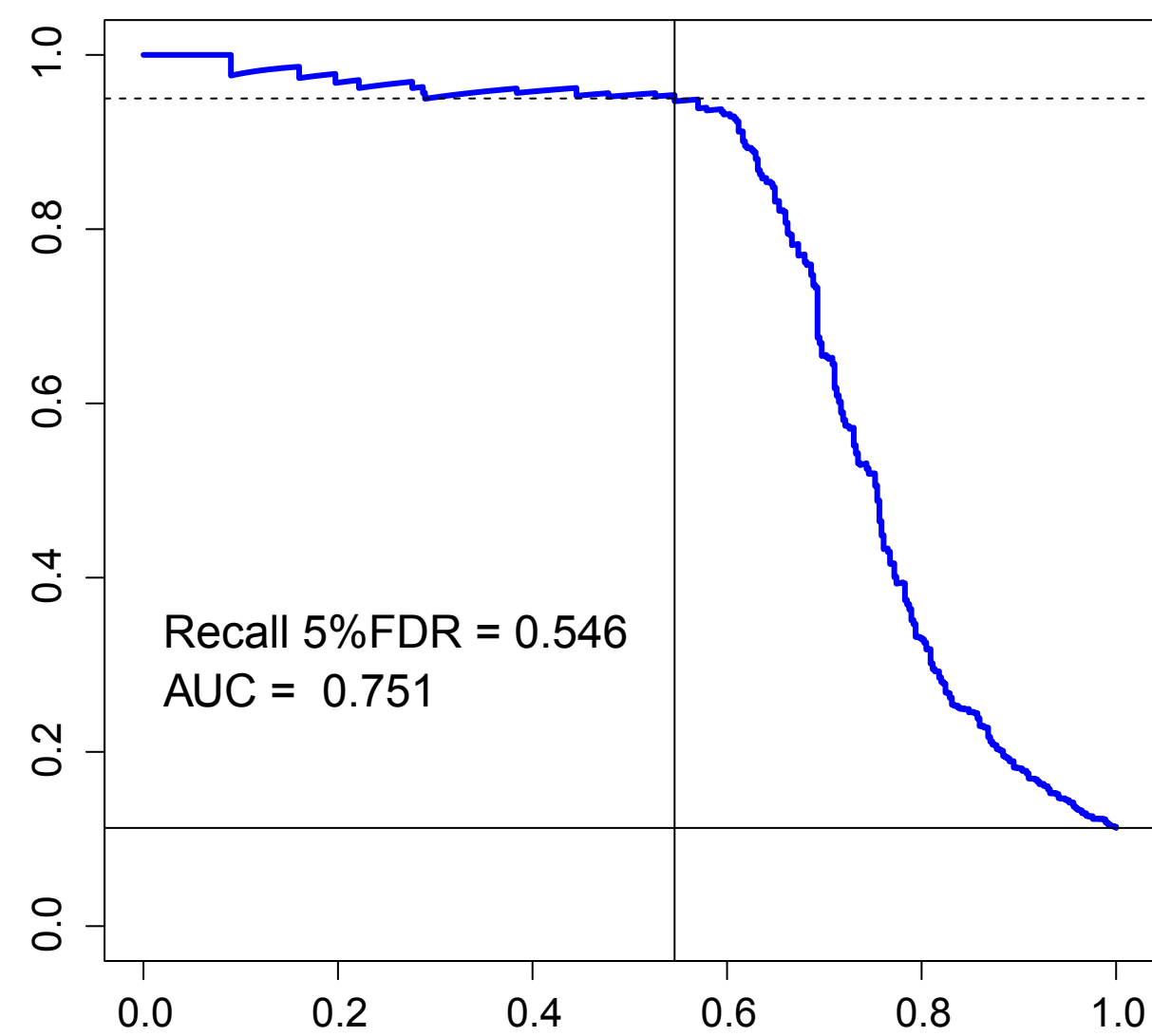**CENtools**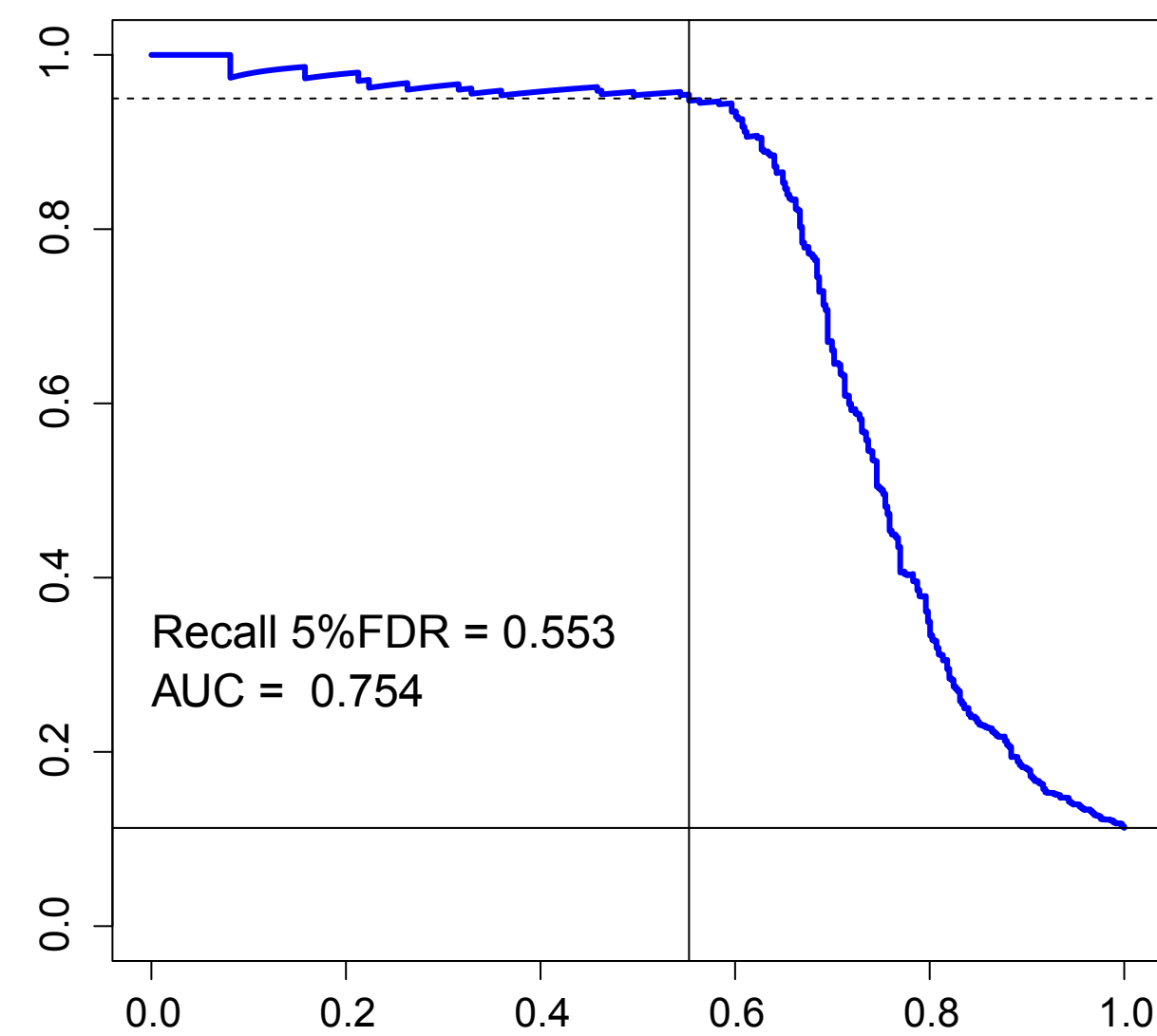**curated\_Hart2014**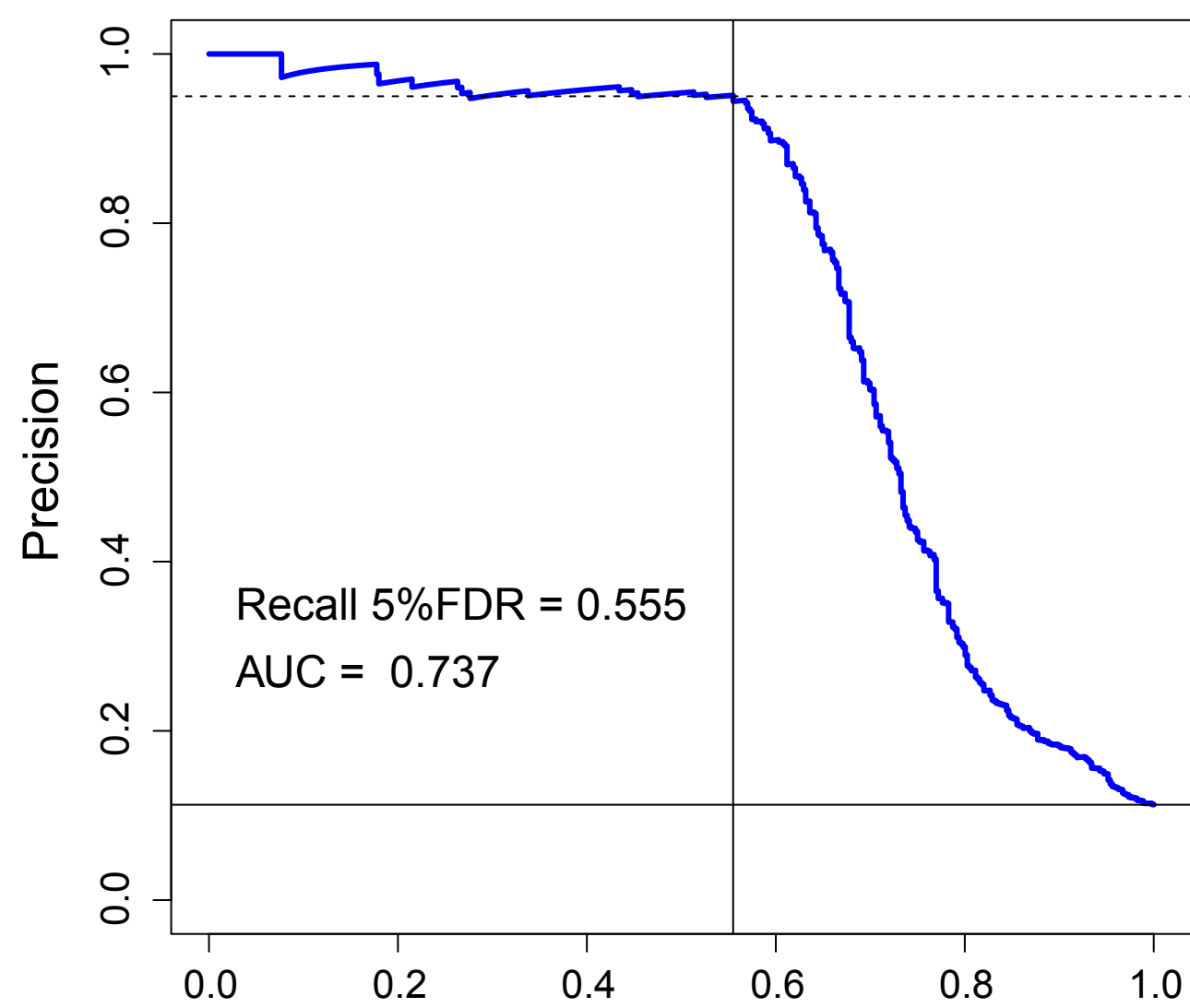**Hart2014**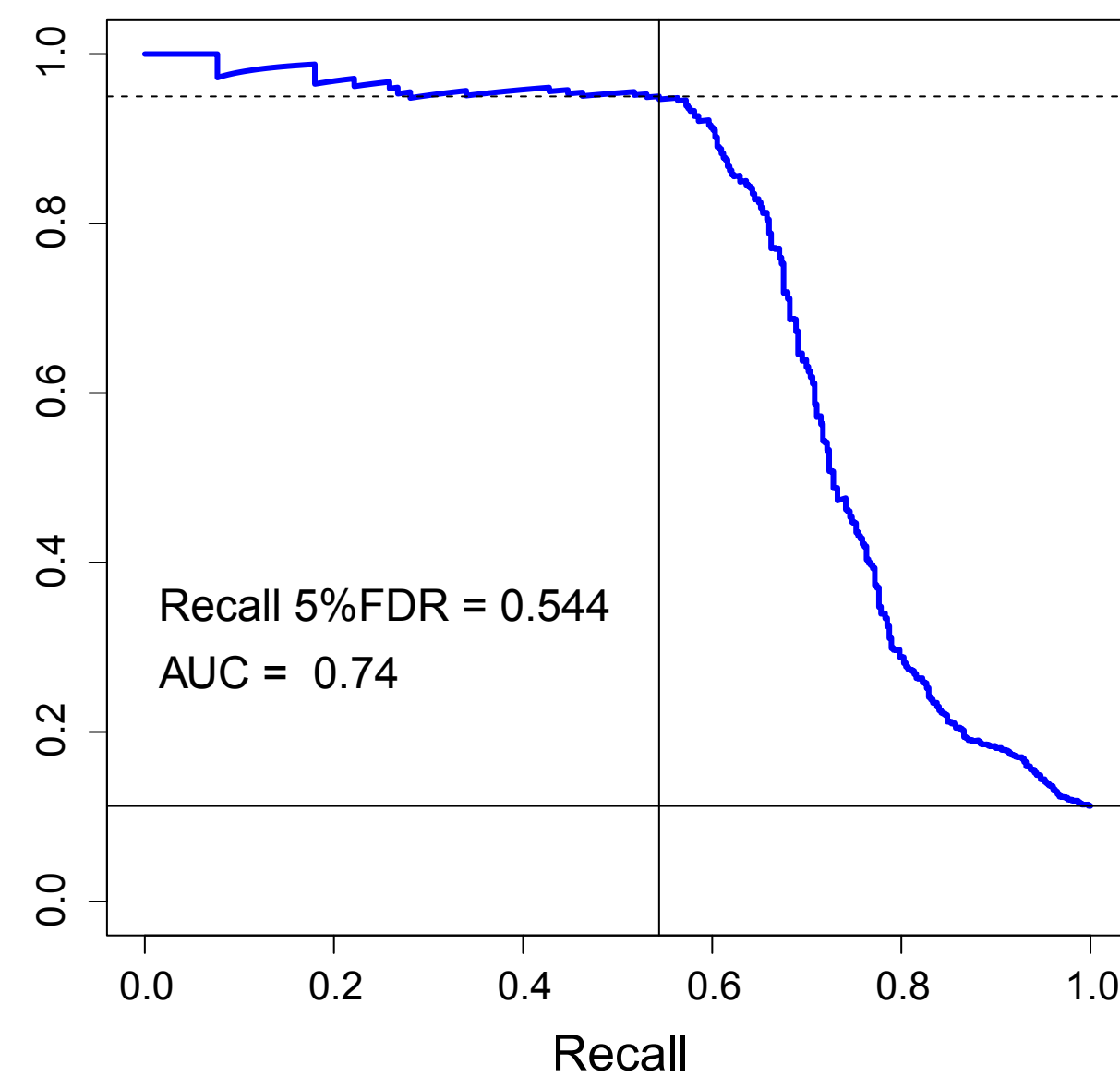**Hart2017**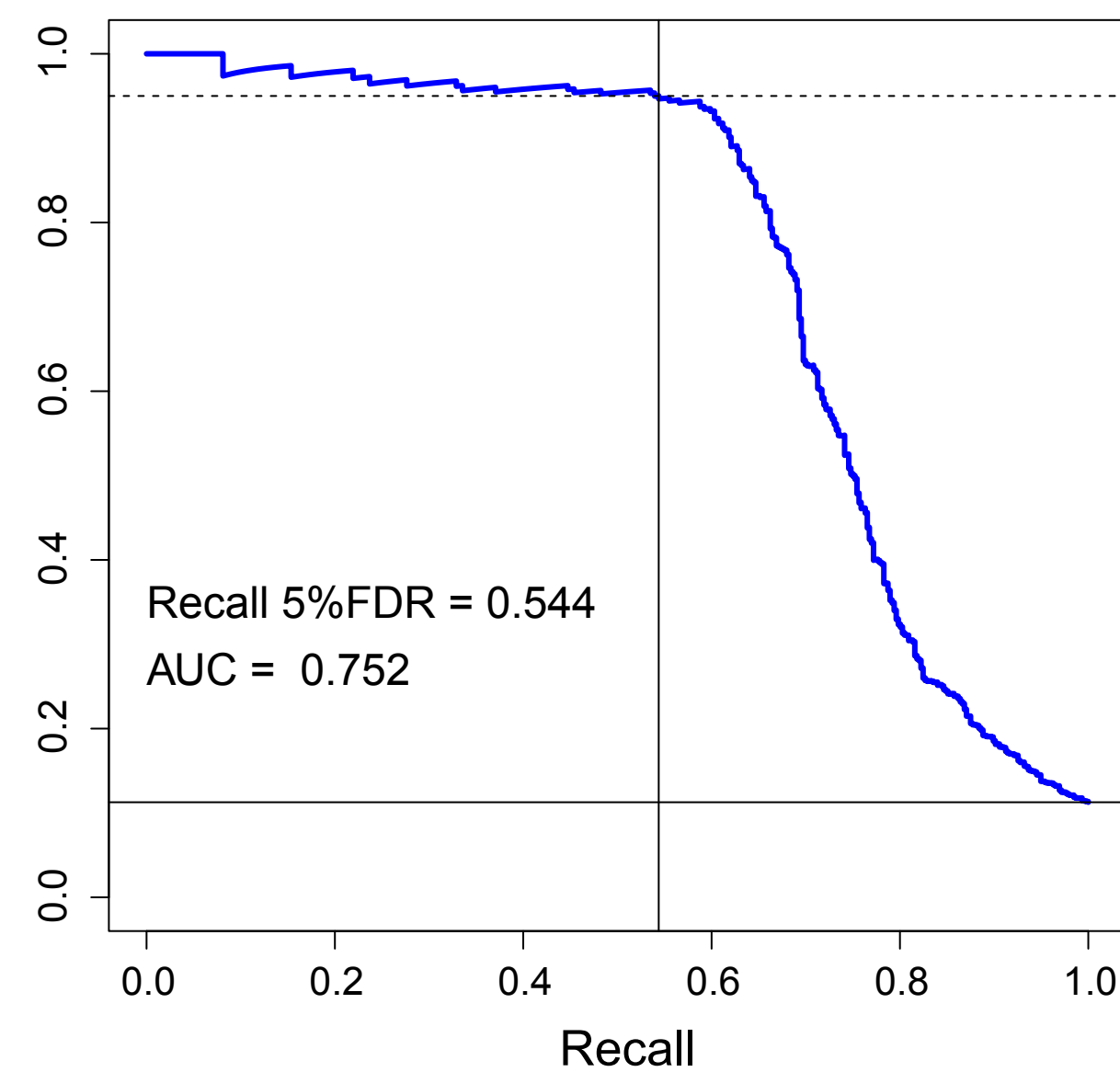**Sharma2020**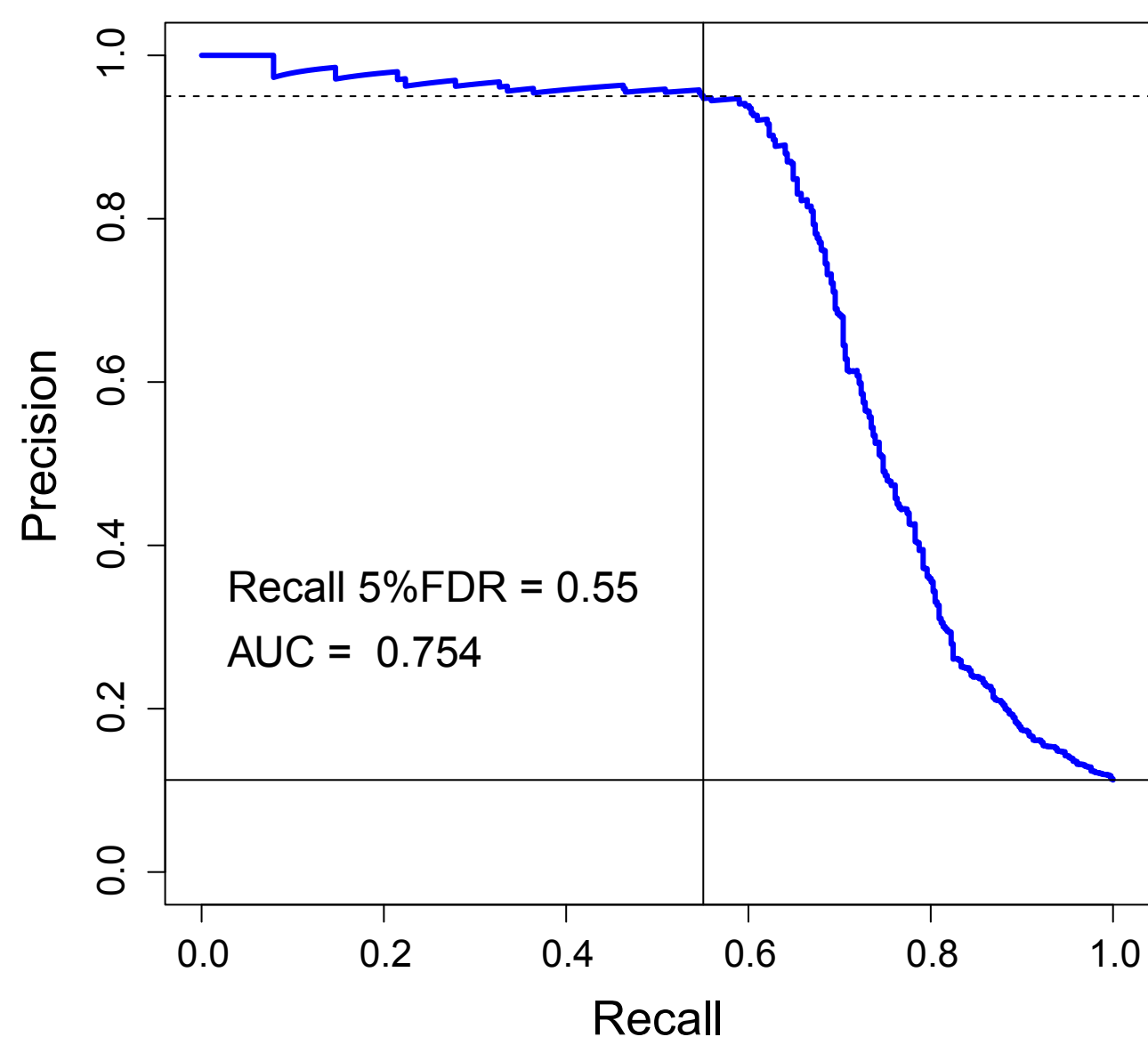

Supplement: Supplementary file 10 — Additional File 10: Fig. S6. Precision-recall curves of oncogene addictions versus not-expressed oncogenes yielded by rank-based classifiers based on bayesian factors computed with BAGEL when using the compared sets of CFGs as positive training sets. [file 12864_2021_8129_MOESM10_ESM.pdf]
