# Supplementary material for: CoRe: a robustly benchmarked R package for identifying core-fitness genes in genome-wide pooled CRISPR-Cas9 screens
Source: BMC Genomics. 2021 Nov 17;22:828. doi: 10.1186/s12864-021-08129-5 (PMC8597285; doi:10.1186/s12864-021-08129-5)
Supplement: Supplementary file 11 — Additional File 11: Additional methods and documentation. [file 12864_2021_8129_MOESM11_ESM.pdf]

Additional File 11: Additional methods and documentation

for

CoRe: a robustly benchmarked R package for identifying core-fitness genes in genome-wide pooled CRISPR-Cas9 screens

Alessandro Vinceti[1], Emre Karakoc[2], Clare Pacini[2], Umberto Perron[1], Riccardo Roberto De Lucia[1], Mathew J. Garnett[2], Francesco Iorio[1,2,†]

[1] Human Technopole, Milano, Italy
[2] Wellcome Sanger Institute, Wellcome Genome Campus, Hinxton, Cambridge, UK
[†]Correspondence: francesco.iorio@fht.org

Additional methods
- Source of reference essential and non-essential genes
- DepMap dataset acquisition and pre-processing
- CEN-tools Logistic Regression execution
- Execution of ADaM
- Execution of FiPer variants
- Benchmark of pan-cancer core-fitness gene sets
- Characterisation of novel pan-cancer core-fitness sets
- Benchmark using an independent cancer dependency dataset
- Retrieval of oncogenetic addictions as bayesian factor templates
- Hardware and software used to compare computational performances

Additional documentation
- Running CENtools logistic regression and clustering

Additional References

Additional methods

Source of reference essential and non-essential genes

The Hart2014 set (used as reference sets of prior known essential genes) and the BAGEL non-essential set (used as reference sets of prior known non-essential genes) are introduced in [1] and are derived from a collection of shRNA screens across 72 breast, ovarian and pancreatic human cancer cell lines [2]. We have further curated these sets by removing high confidence cancer driver genes as described in [3]. The Hart2017 (used to train the logistic regression model in [4] together with the BAGEL non-essential set) set was derived in [5] from a reanalysis of 17 genome-wide CRISPR-Cas9 knock-out employing three different CRISPR libraries [6–9]. All these sets are independent from the DepMap dataset used in our study and described below.

DepMap dataset acquisition and pre-processing

We downloaded the latest version of the integrated Sanger and Broad essentiality matrix processed with CERES [10], from the DepMap portal (https://www.depmap.org/broad-sanger/integrated_Sanger_Broad_essentiality_matrices_20201201.zip). Among the 908 cell lines/columns, we found 51 containing missing values, thus we removed them. We then kept only the cell lines with an associated cancer tissue in the Cell Model Passport (annotation file version 20210326, https://cog.sanger.ac.uk/cmp/download/model_list_20210326.csv.gz), totalling to 855. This step is required to run ADaM tissue-wise. The dataset was then scaled column-wise to have the median of curated BAGEL essential gene fitness scores equal to -1 and the median of curated BAGEL never-essential equal to 0 across all cell lines.
For the execution of ADaM, we binarised the pre-processed CERES dataset considering as essential all genes having a fitness score less than -0.5 in each cell line. For binarising the fitness score matrix, other strategies might be adopted: for example, BAGEL2 [11] can be used for the estimation of gene-level bayesian factors, followed by per-sample scaling by subtracting the 5% FDR threshold between the fitness score distributions of reference sets of essential and non-essential genes, and binarization by assigning 1 to all positive scores, 0 otherwise, as illustrated in [3].

CEN-tools Logistic Regression execution

We downloaded the CEN-tools package [4] from https://gitlab.ebi.ac.uk/petsalakilab/centools/-/tree/master/CEN-tools. In order to decrease the memory burden for the GitHub repository of the CoRe package, we removed all the python modules and data objects that were not directly called by the LR.py and clustering.R

functions, respectively the python script implementing the logistic regression model and the R script performing the subsequent cluster analysis.

In addition, we added a few lines of code to the LR.py script to make it runnable from the command line and assembling data objects on the fly. Particularly, CEN-tools uses a python dictionary in pickle format to specify which genes belong to the true positive set (i.e., curated BAGEL essential) or true negative set (i.e., curated BAGEL never-essential). We seeded both scripts to guarantee reproducibility. All changes applied to the CEN-tools script are detailed in the **Additional Documentation** below.

While executing the logistic regression model implemented by CEN-tools we used the curated BAGEL essential and curated never-essential genes for the training phase [3]. Based on the logistic regression, CEN-tools computes a matrix of continuous probability distributions for each gene being essential across cell lines and discretizes them according to the number of bins specified by the user. We adopted 20 bins as this was the default parameter used in the original CEN-tools run [4]. Subsequently, we normalised the matrix and clustered the genes not included in the training sets through k-means using the Hartigan-Wong algorithm [12], around four centres. The silhouette method identified four as the optimal number of clusters according to their probability essentiality profiles: core essential, context-specific, rare-context-specific, and never-essential. The core essential genes are characterized by the highest value of silhouette width, thus we used them for the downstream benchmarking.

Tissue specific execution of ADaM

ADaM takes as input a binarised matrix of fitness essentiality scores. For the identification of tissue CFGs, only the N cell lines that are part of the same cancer tissue/type T are selected. ADaM then computes a fuzzy intersection I_n composed of genes exerting a significant depletion in at least n cells out of N. The optimal number of cell lines n^* in which a gene should be essential in order to be predicted as core-fitness essential is then obtained in a semi-supervised manner, as it follows. For each fuzzy intersection I_n from $n = 1, \ldots, N$, a true positive rate ($TPR(n)$) is computed considering a set E of prior known essential genes as true positives, as it follows:

$$TPR(n) = |E \cap I_n| / |E \cap G|$$

where G indicates the whole set of screened genes.

In addition, ADaM computes the $\log_{10}$ odd ratio between the observed I_n and its expectation value $Ex(I_n)$:

$$OR(n) = log_{10}(I_n / Ex(I_n)).$$

$Ex(I_n)$ is estimated by shuffling the binary matrix column-wise 1,000 times, i.e. preserving the number of essential genes for every cell line. Then $Ex(I_n)$ is defined as the average value of the I_n cardinalities:

$$Ex(I_n) = \frac{1}{1000}\sum_{i=1}^{1000} |I_n|.$$

The threshold n^* corresponds to the minimal number of cell lines n whose I_n provides the trade-off between the two monotonic functions, $TPR(n)$ being inversely proportional to n and $OR(n)$ being directly proportional to n.

This is implemented by the wrapper function CoRe.CS_ADaM that subsets the dataset by taking only the cell lines included in the cancer tissue/type of interest using the Cell Model Passport [13] annotation file. We used the annotation file version 20210326.

We executed ADaM using the CERES binarised DepMap dataset and using the curated BAGEL essential genes as reference true positives. We set the number of random trials for the generation of the null model to 1,000 and ran the algorithm only on those cancer tissues with at least 15 cell lines available as detailed in [3].

Execution of FiPer variants

We used the function CoRe.FiPer of CoRe. Initially, the method computes a gene-wise cell line ranking R_{CL}, where for each gene g it ranks every cell line cl according to the fitness essentiality score of g in cl. It also computes a cell-wise gene ranking R_G, where for each cell line it ranks every gene according to the fitness essentiality of g in cl. Then the package implements four different variants of the fitness percentile method:

- Fixed = a distribution of gene fitness-rank-positions in their most dependent n^{th} (determined by the percentile parameter, default is 90th) percentile cell line is used in the subsequent step;
- Average = a distribution of average gene fitness-rank-positions across cell lines at or over the nth percentile of most dependent (determined by the percentile parameter, default is 90th) cell lines is used in the subsequent step;
- Slope = for each gene g, a linear model is fit on the sequence of gene fitness-rank-positions across all cell lines sorted according to their dependency on g, then a distribution of models' slopes is used in the subsequent step;

- AUC = for each gene *g*, the area under the curve (AUC) resulting from considering the sequence of gene fitness-rank-positions across all cell lines sorted according to their dependency on g is used in the subsequent step.

Each FiPer variant outputs a discrete distribution of gene fitness-rank-positions. A gaussian kernel estimator is applied to compute a continuous distribution. The kernel density estimator uses a default bandwidth defined as 0.9 times the minimum of the standard deviation and the interquartile range divided by 1.34 times the sample size to the negative one-fifth power. The distribution is bimodal, and the rank threshold corresponds to the local minimum. All genes having a fitness-rank-position lower than the threshold are classified as CEGs. In the benchmarking, we assessed all the four variants on the full pre-processed CERES matrix to identify the pan-cancer CEGs.

Benchmark of pan-cancer core-fitness gene sets

We designed a baseline predictor to assess the sets of pan-cancer core-fitness genes computed by each method. This was defined by considering core-fitness those genes essential in at least *n* cell lines, for all possible *n*.

Next we assemble sets of priori known positive/negative control essential genes. For the positive controls we collected signatures of genes involved in fundamental biological processes and universally essential genes: such as genes coding for ribosomal proteins, RNA polymerases, histones, or genes involved in DNA replications, etc. curated in [14] and [15] from MsigDB [16]. As negative controls we assembled a set of genes never expressed (fragments per kilobase of transcript per million mapped reads (FPKM) < 0.1) in more than 1,000 human cancer cell lines (from the Cell Model Passports [13]), or whose fitness signal across hundreds of cell lines has a t-skewed normal distribution (according to the normLRT score introduced and applied to an independent shRNA-based cancer dependency dataset in [17]) and it is statistically associated with a genomic marker [15]. Excluding genes included in at least one of the training sets yielded a final set of 408 positive controls and 7,767 negative controls. Of these, 265 positive controls and 555 negative controls were included in the DepMap dataset.

The metrics derived from the baseline predictor were used to assess each CFG and CEG set. We considered both novel hits, namely the tested sets stripped out of the BAGEL genes used in the training phase of the two CEN-tools runs (i.e. the Hart2017 set, the BAGEL non-essential genes [1], curated BAGEL essential and never-essential genes [3]), as well as in their entirety. The recall of each set was normalised by the maximum recall achieved by the baseline predictor and so was done for the false positive rate. The two coordinates associated with each set were used to perform a cubic spline interpolation [18] and evaluate

the balance between the normalised recall and false positive ratios according to the size of the set.

Next, we computed the thresholds required by the baseline predictor to attain the recalls observed by all tested methods and corresponding false positive rates.

Characterisation of novel pan-cancer core-fitness sets

To identify biologically grounded novel pan-cancer CF sets, we considered the gene families found enriched across all the predicted sets. For every set, we performed a hypergeometric test for each gene family with at least one gene in the CFG set of interest, following the formula:

$$p_{x,f}(k) = Pr(x > k) = \sum_{i=k+1}^{K} \frac{\binom{K}{i}\binom{N-K}{n-i}}{\binom{N}{n}}$$

where *p* is the associated probability value of having more genes than observed *k* for a given family *f* in the CFG set under consideration, *K* is the total number of genes in the CFG set associated to any functional family, *N* is the total number of screened genes in the DepMap dataset preprocessed matrix associated to any functional family, *n* is the total number of genes belonging to f and found either in the CFG set or the remaining screened genes.

The *p*-values were then pooled and corrected set-wise using the Benjamini-Hochberg procedure. Gene families with an adjusted *p*-value < 0.05 in each CFG set were deemed significant. The significantly enriched families in common across the supervised methods (i.e., ADaM and CEN-tools in both instances, including also the Hart2014 and Hart2017 sets) were classified as always enriched and the pooled CFG sets used as ground truth. Particularly, we computed the exclusive CEGs in the FiPer AUC set belonging to the always enriched families that were not found in the ground truth. These CEGs were classified as novel hits.

In addition, we repeated the analysis assembling the significantly enriched families in common across the unsupervised methods (i.e. the four variants of the fitness percentile method plus the FiPer consensus set) and showed that unsupervised methods have higher sensitivity in identifying families derived from late time-point essential gene sets [19].

Benchmark using an independent cancer dependency dataset

We downloaded the DEMETER v6 04/20 cancer dependency data derived from genome-wide RNAi screens [20] (available at

https://ndownloader.figshare.com/files/11489669). This dataset was scaled column-wise in order to have the median of curated BAGEL essential gene fitness scores equal to -1 and the median of curated BAGEL never-essential equal to 0 across all cell lines.

For each tested CFG/CEG set, we derived the median DEMETER fitness scores for every gene across cell lines. In addition, we also derived the median DEMETER fitness scores of the genes included in the CFG sets predicted by the baseline classifier, at the observed TPRs, and computed the normalised scores across sets.

Retrieval of oncogenetic addictions as bayesian factor templates

We ran BAGEL v115 on the Sanger release 1 cancer dependency dataset (downloaded from: https://score.depmap.sanger.ac.uk/downloads) processed with CRISPRcleanR [14] (shown in [15] to better preserve context-specific essentialities than CERES). As a positive training gene set we used each of the tested CFG sets, in turn, whereas as a negative training gene set we used the curated BAGEL never-essential genes. This led to seven different Bayesian factor (BF) matrices. Each was scaled cell-wise by subtracting to each gene the 5% false discovery rate (FDR) threshold computed between the BF scores of the two training distributions, for comparability.

Next, we defined a set of cell line specific true positives and negatives to assess the ability of the templates in recapitulating oncogene addictions. First, we assembled a binary matrix summarizing the status of pan-cancer Cancer Functional Events (CFEs) across Sanger cell lines, namely somatic mutations, copy number alterations, and hypermethylation. The binary matrix was then subset to include only genes unambiguously classified as oncogenes in the catalog of driver genes release 2020.02.01 from the IntOGen database. In addition, copy number gains in genomic segments containing ERBB2 or EGFR or KRAS or MYC or MYCN (typically copy number amplified oncogenes in different cancer types), were considered as positive events too. Secondly, we assembled an additional binary matrix where we deemed as positive events oncogenes not expressed in a cell line. We considered oncogenes only instead of including all not expressed genes to avoid unbalanced control sets, favouring the negative controls. By combining the two binary matrices, we obtained three classes:

- Positive instances constituted by oncogenes mutated and expressed in a cell line;
- Null instances constituted either by wild-type and expressed or mutated and not expressed oncogenes in the cell line;
- Negative instances constituted by wild-type and not expressed oncogenes in the cell line.

The positive and negative instances were used on the BF score of each template to assess the area under precision-recall curve and the recall at fixed percentages of FDRs.

Hardware and software used to compare computational performances

All the analyses were performed on a MacOS laptop with a 2.3 GHz Quad-Core Intel Core i7 processor and 16 GB RAM. The operating system was Big Sur v11.2.3 (20D91). The software was executed in the RStudio IDE v1.3.1073 with x86_64-apple-darwin17.0 platform and R programming language v4.0.2, python scripts were executed using python v3.9.1. For all the methods shown in Table 1 below but ADaM, we used the quantitative pre-processed CERES matrix. The matrix consisted of 17,846 genes and 855 cell lines containing gene fitness scores. Instead, ADaM used a binarized version of the matrix as explained in the previous section. The binary matrix consisted of 8,496 genes, considering genes classified as essential in at least one cell line, and 820 cell lines, considering cell lines from a cancer tissue with at least 15 cell lines in total.

In addition, we executed the CoRe methods and CEN-tools on an ASUS laptop with a 1.80 GHz Quad-Core Intel Core i7 processor and 8 GB RAM. The laptop resources were partitioned 50/50 following a dual-boot configuration, presenting on one end Ubuntu 16.04 LTS as operating system, and Windows 10 on the other. The methods were tested on both operating systems.

Additional documentation

Running CENtools logistic regression and clustering

First, we downloaded the CENtools folder from https://gitlab.ebi.ac.uk/petsalakilab/centools/-/tree/master/CENtools. The files in the prediction subfolder were moved to the parent directory and the subfolder subsequently removed. We also removed the cluster_annotation.py. Indeed, this script should be run after the R script clustering.R, however, we don't need it for cluster object serialisation, since we saved the CENtools core essential genes as R data. Then we removed the analysis folder, since it was not used in the logistic regression.

We also downloaded the data folder from the main CENtools repository (https://gitlab.ebi.ac.uk/petsalakilab/centools/-/tree/master/) and moved it in the CENtools folder. For the purpose of our analysis, we removed unnecessary data to have a more lightweight folder:

- All data objects in the objects subfolder. Here, we have a pickle dictionary defining for each screened gene in the dataset whether it belongs to the positive training set (i.e. curated BAGEL essential) or the negative training set (i.e. curated BAGEL

non-essential). To save memory, the notebook creates this object on the fly and then removes it when done.

- In the curated_data subfolder, we removed all the heavy fitness score datasets to save memory. The notebook saves the CERES_scaled_depFC.csv dataset on the fly, processed with the pipeline illustrated in the CoRe paper, and removes it when done.

We had to make some adjustments in the logistic regression script (i.e. LR.py) to make it runnable from the command line by adding a few lines of code at the end. We also commented out the figure saving instances. In this way, the script just creates and saves the pickle dictionary on the fly, executes the logistic regression model with the chosen parameters and returns only the output file of interest in a subfolder defined 'prediction_output/INTEGRATED/'. Furthermore we performed the following operations in the LR.py script:

- The code was seeded in order to guarantee reproducibility.

- In lines 44 and 332, we replaced:

  if "prediction_output" not in os.listdir(path): os.system("mkdir %s" %path + "prediction_output")

  os.system("mkdir %s" % prediction_path + args["PROJECT"])

  With:

  if "prediction_output" not in os.listdir(path): os.mkdir(path + "prediction_output")

  os.mkdir(prediction_path + args["PROJECT"])

  The outcome is the same (i.e. the creation of a subfolder in the designated path), however, this adjustment was needed in order for the interpreter to not throw an error.

- In line 392, we replaced:

  bin_df = pandas.DataFrame.from_dict(bin_vector_dicts).T

  With:

  geneKeys = bin_vector_dicts.keys()

  bin_df = numpy.array([bin_vector_dicts[i][bin_number][bin_number][j] for i in geneKeys for j in range(0, bin_number)])

  bin_df = numpy.reshape(bin_df, (len(geneKeys), 20))

  bin_df = pandas.DataFrame(bin_df, index=geneKeys)

In order to save the output as a matrix that can be easily read by the R script clustering.R.

- In addition, in line 393 the typo *"bin_number"* was replaced with *"BIN_NUMBER"*.

In the paths.py script, line 4:

path = os.getcwd() + "/venv/"

Was replaced with:

path = os.getcwd() + "/CENtools/"

This adjustment makes the relative pathways available for the jupyter notebook.

Other modifications were done on the data_preparation subfolder. First, objects_.py was renamed as objects.py to make it executable from other scripts. Furthermore, we performed the following operations in the curation.py script:

- In lines 22 and 23:

  BEG = [line.strip() for line in open(curated_data_path + "Bagel_Essential_Genes.txt").readlines()]

  BNEG = [line.strip() for line in open(curated_data_path + "Bagel_Non_Essential_Genes.txt").readlines()]

  Were replaced with:

  BEG = [line.strip() for line in open(curated_data_path + "Curated_Bagel_Essential_Genes.txt").readlines()]

  BNEG = [line.strip() for line in open(curated_data_path + "Curated_Bagel_Non_Essential_Genes.txt").readlines()]

  This allowed to set the curated BAGEL essential and non-essential genes as default sets used in the training phase.

- In line 75:

  integrated_cor_fc = pandas.read_csv(curated_data_path + "integrated_cor_FC_essentiality.csv", index_col=0)

  Was replaced with:

  integrated_cor_fc = pandas.read_csv(curated_data_path + "CERES_scaled_depFC.csv", index_col=0)

This allowed to set our preprocessed CERES dataset as default when performing the logistic regression.

Finally, in the R script clustering.R we commented out some parts of the code and made the function *"ClusterEssentiality"* returning just the string character vector of predicted core essential genes. Here too the code was seeded to guarantee reproducibility.

Additional References

1. Hart T, Brown KR, Sircoulomb F, Rottapel R, Moffat J. Measuring error rates in genomic perturbation screens: gold standards for human functional genomics. Mol Syst Biol. 2014;10:733.

2. Marcotte R, Brown KR, Suarez F, Sayad A, Karamboulas K, Krzyzanowski PM, et al. Essential gene profiles in breast, pancreatic, and ovarian cancer cells. Cancer Discov. 2012;2:172–89.

3. Behan FM, Iorio F, Picco G, Gonçalves E, Beaver CM, Migliardi G, et al. Prioritization of cancer therapeutic targets using CRISPR-Cas9 screens. Nature. 2019;568:511–6.

4. Sharma S, Dincer C, Weidemüller P, Wright GJ, Petsalaki E. CEN-tools: an integrative platform to identify the contexts of essential genes. Mol Syst Biol. 2020;16:e9698.

5. Hart T, Tong AHY, Chan K, Van Leeuwen J, Seetharaman A, Aregger M, et al. Evaluation and Design of Genome-Wide CRISPR/SpCas9 Knockout Screens. G3 . 2017;7:2719–27.

6. Koike-Yusa H, Li Y, Tan E-P, Velasco-Herrera MDC, Yusa K. Genome-wide recessive genetic screening in mammalian cells with a lentiviral CRISPR-guide RNA library. Nat Biotechnol. 2014;32:267–73.

7. Hart T, Chandrashekhar M, Aregger M, Steinhart Z, Brown KR, MacLeod G, et al. High-Resolution CRISPR Screens Reveal Fitness Genes and Genotype-Specific Cancer Liabilities. Cell. 2015;163:1515–26.

8. Steinhart Z, Pavlovic Z, Chandrashekhar M, Hart T, Wang X, Zhang X, et al. Genome-wide CRISPR screens reveal a Wnt-FZD5 signaling circuit as a druggable vulnerability of RNF43-mutant pancreatic tumors. Nat Med. 2017;23:60–8.

9. Wang T, Birsoy K, Hughes NW, Krupczak KM, Post Y, Wei JJ, et al. Identification and characterization of essential genes in the human genome. Science. 2015;350:1096–101.

10. Meyers RM, Bryan JG, McFarland JM, Weir BA, Sizemore AE, Xu H, et al. Computational correction of copy number effect improves specificity of CRISPR-Cas9 essentiality screens in cancer cells. Nat Genet. 2017;49:1779–84.

11. Kim E, Hart T. Improved analysis of CRISPR fitness screens and reduced off-target effects with the BAGEL2 gene essentiality classifier. Genome Med. 2021;13:2.

12. Hartigan JA, Wong MA. Algorithm AS 136: A K-Means Clustering Algorithm. J R Stat Soc Ser C Appl Stat. 1979;28:100–8.

13. van der Meer D, Barthorpe S, Yang W, Lightfoot H, Hall C, Gilbert J, et al. Cell Model

Passports—a hub for clinical, genetic and functional datasets of preclinical cancer models. Nucleic Acids Res. 2019;47:D923–9.

14. Iorio F, Behan FM, Gonçalves E, Bhosle SG, Chen E, Shepherd R, et al. Unsupervised correction of gene-independent cell responses to CRISPR-Cas9 targeting. BMC Genomics. 2018;19:604.

15. Pacini C, Dempster JM, Boyle I, Gonçalves E, Najgebauer H, Karakoc E, et al. Integrated cross-study datasets of genetic dependencies in cancer. Nat Commun. 2021;12:1661.

16. Subramanian A, Tamayo P, Mootha VK, Mukherjee S, Ebert BL, Gillette MA, et al. Gene set enrichment analysis: a knowledge-based approach for interpreting genome-wide expression profiles. Proc Natl Acad Sci U S A. 2005;102:15545.

17. McDonald ER 3rd, de Weck A, Schlabach MR, Billy E, Mavrakis KJ, Hoffman GR, et al. Project DRIVE: A Compendium of Cancer Dependencies and Synthetic Lethal Relationships Uncovered by Large-Scale, Deep RNAi Screening. Cell. 2017;170:577–92.e10.

18. Hall CA, Meyer WW. Optimal error bounds for cubic spline interpolation. J Approx Theory. 1976;16:105–22.

19. Tzelepis K, Koike-Yusa H, De Braekeleer E, Li Y, Metzakopian E, Dovey OM, et al. A CRISPR Dropout Screen Identifies Genetic Vulnerabilities and Therapeutic Targets in Acute Myeloid Leukemia. Cell Rep. 2016;17:1193–205.

20. McFarland JM, Ho ZV, Kugener G, Dempster JM, Montgomery PG, Bryan JG, et al. Improved estimation of cancer dependencies from large-scale RNAi screens using model-based normalization and data integration. Nat Commun. 2018;9:4610.
